# ﻿Parallel developments in floral adaptations to obligate moth pollination mutualism in tribe Phyllantheae (Phyllanthaceae)

**DOI:** 10.3897/phytokeys.225.99506

**Published:** 2023-05-02

**Authors:** Peter C. van Welzen, Esmée Winkel, Roderick W. Bouman

**Affiliations:** 1 Naturalis Biodiversity Center, PO Box 9517, 2300 RA Leiden, Netherlands; 2 Institute of Biology Leiden, Leiden University, PO Box 9505, 2300 RA Leiden, Netherlands; 3 Hortus botanicus Leiden, Leiden University, PO Box 9500, 2300 RA Leiden, Netherlands

**Keywords:** *Breynia*, *Cicca*, *Dendrophyllanthus*, *Epicephala* moths, *Glochidion*, *Kirganelia*, morphological adaptation, *Phyllanthus*

## Abstract

Several groups within tribe Phyllantheae (Phyllanthaceae) formed, independently, an (obligate) pollination mutualism with *Epicephala* moths, which originally had been parasitic. In this pollination system, female moths actively collect pollen from staminate flowers and deposit it on the stigma of pistillate flowers, after which they place at least one egg in or against the ovary. The high pollination rate makes the system beneficial for the plants, whereas the larvae are provided with food (part of the developing seeds) and some protection against predation. Qualitative comparisons are made between non-moth-pollinated lineages, used as outgroups and various, independently moth-pollinated Phyllantheae clades, used as ingroups, thereby looking for parallel developments. The flowers of both sexes of various groups display similar, convergent morphological adaptations to the pollination system, likely to secure the obligate relationship and to improve efficiency. Sepals in both sexes, free or partly to highly connate, are commonly upright and form a narrow tube. The staminate flowers often have united, vertical stamens with the anthers along the androphore or on top of the androphore. Pistillate flowers generally reduce the stigmatic surface, either by making the stigmas shorter or by uniting them into a cone with a small opening at the top for pollen deposition. Less obvious is the reduction of the stigmatic papillae; these are often present in non-moth-pollinated taxa, but absent in the moth-pollinated species. The most diverging, parallel adaptations to moth pollination are currently found in the Palaeotropics, whereas in the Neotropics, some groups continue to also be pollinated by other insect groups and are morphologically less changed.

## ﻿Introduction

Obligate pollination mutualisms between insects and plants have developed in several plant families and they provide interesting study systems for reciprocal morphological evolution and adaptation between the partners. Kato and Kawakita (2017, chapter 13) provide a nice overview of the various plant groups and their pollinators. Three examples where the relationship between both parties is obligate and where a proliferation of co-speciation occurred, are famous: figs and fig-wasps, *Yucca* L. and *Yucca* moths and leafflower and leafflower moths (Kato and Kawakita 2017). The latter group forms the main topic of this paper, particularly tribe Phyllantheae Dumort. (Hoffmann et al. 2006). The tribe mainly consists of the large (paraphyletic) *Phyllanthus* L. (Wurdack et al. 2004; Samuel et al. 2005; Kathriarachchi et al. 2005, 2006), a clade that also includes *Breynia* J.R.Forst. & G.Forst., *Glochidion* J.R.Forst. & G.Forst. and *Synostemon* F.Muell.

**Figure 1.**
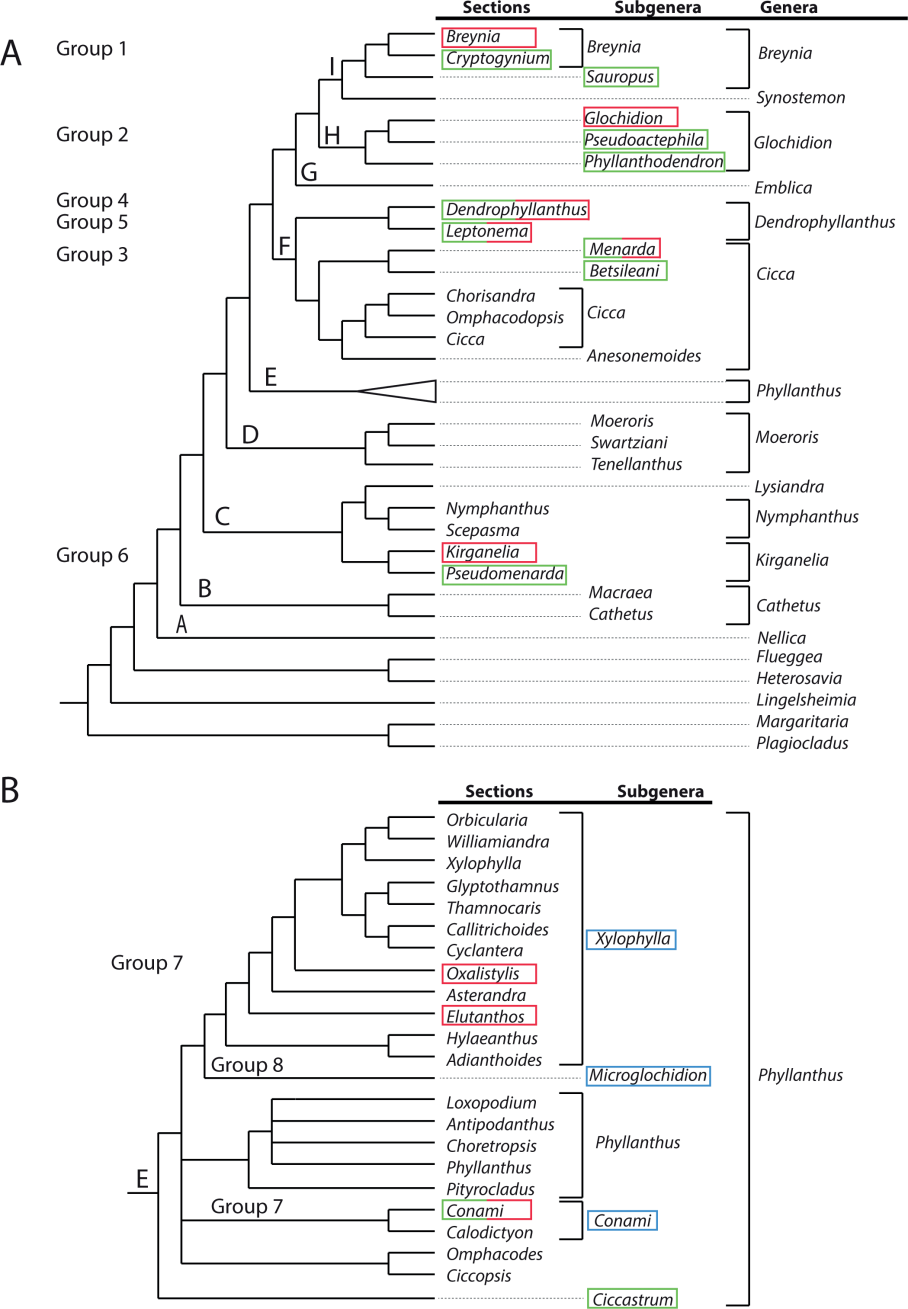
Summary of the Bayesian phylogeny and new classification of tribe Phyllantheae, based on the markers ITS, PHYC, *accd*, *trnSG* and matK; Bouman et al. (2022)) **A** the new classification runs from right to left **B** shows the detail of the classification and phylogeny of *Phyllanthus* L. Red boxes in **A** indicate the six groups with moth pollination; in green are the clades used as outgroups with which they are morphologically compared. Boxes with green and red represent clades with first splitting off the non-moth-pollinated species and in the top part of the clade the moth-pollinated ones. In blue boxes (**B**), two South American groups in *Phyllanthus* are highlighted, which, based on morphology, might also have moth pollination.

Kato et al. (2003) were the first to report the obligate pollination mutualism between the Phyllanthaceae plants (genus *Glochidion*) and the *Epicephala* moths. Most active research on this relationship was done by Kawakita and Kato and their results are summarised in their book (Kato and Kawakita 2017). It is now obvious that the relationship between Phyllantheae and moths originated at least six times independently (Kawakita and Kato 2009), which is confirmed by the phylogeny of Bouman et al. (2021), see the (partly) red-boxed groups in Fig. 1A, likely also several times in the genus *Phyllanthus* (Fig. 1B, see also Discussion). The morphology of staminate and pistillate flowers changed and adapted accordingly (for example, see Zhang et al. (2012); Yang and Li (2018)). The female moths of mutualistic species actively collect pollen from staminate flowers, whereby the sensilla or setae (hairs) on the proboscis sweep up the pollen (these hairs are absent in male moths and in (cheating) moths with no mutualistic relationship; Kato and Kawakita (2017); Yang and Li (2018); Wang et al. (2020)). The female moths then place the pollen on the stigmas of the pistillate flowers and finally place an egg either between the calyx and the ovary, or in the ovary by drilling through the stigma or through the ovary wall (Kato et al. 2003). This system provides the plants with a (usually) specific pollinator and, in return, the moths gain shelter and food for the developing larvae, which feed on some of the developing seeds. Seed consumption is variable and indicative of variations in the relationship between plant and pollinator; in some cases, only some of the seeds are consumed, while in others all can be eaten (Zhang et al. 2012).

*Epicephala* belongs to the family Gracillariidae, a family of micro-lepidoptera with ca. 100 genera and 2000 species (see Kato and Kawakita (2017): chapter 5, for a good overview and references). Most of them are leaf miners, which sometimes create galls. A few are plant-borers that attack seeds or other parts of the plant. *Epicephala* is part of the generally parasitic leaf-borer group, which has been found to occur also in tribe Phyllantheae (Luo et al. 2011). The phylogeny of *Epicephala* (Kato and Kawakita 2017: fig. 5.3) shows (disputably) *Conopomorphaflueggella* Li, 2011, as outgroup to the *Epicephala* clade. The first group to split off is the New Caledonian group (Kato and Kawakita 2017: fig. 5.3: clade 7). The species in this group are still seed parasites (like *C.fluegella*) (Hu et al. 2011) and lack the sensilla and ovipositor. The species in the remaining six clades have sensilla and an ovipositor and developed the mutualistic pollination syndrome. The exception is clade 2 (Kato and Kawakita 2017: fig. 5.3), where the moths returned to parasitism in the herbaceous *Phyllanthus* species. *Conopomorpha* and clade 7 (Kato and Kawakita 2017: fig. 5.3) are still seed predators, whereby seeds possibly survive due to a high mortality rate amongst the larvae. *Conopomorphaflueggella* feeds on the seeds of *Flueggea* Willd. *Flueggea* is sister to *Phyllanthus* sensu lato (Wurdack et al. 2004; Bouman et al. 2021) and did not develop pollination mutualism. *Flueggea* has very open flowers, whereas the moth-pollinated species generally show more closed flowers. Similarly, seed predation has only been found in developing fruits in species of PhyllanthussubgenusMacraea (Wight) Jean F.Brunel and not in flowers that were yet to be pollinated, which suggests a solely parasitic relationship (Kato and Kawakita 2017). With *Breynia* and (former) *Sauropus* Blume (now subsumed in *Breynia*), it was obvious that both genera were distinguished because of morphological differences in the flowers, seemingly caused by moth pollination (van Welzen 2003). A similar difference seems to be present between *Glochidion* and Phyllanthussubgen.Phyllanthodendron (Hemsl.) G.L.Webster.

The aims of this paper are: 1. to show the qualitative morphological changes in the various flowers of the different plant groups associated with the pollination mutualism by *Epicephala* moths in comparison with their non-mutualistic relatives and 2. to see if there are parallel developments in the morphological adaptations of the (confirmed) six different groups of plants that are now pollinated by the moths, with also a consideration of possibilities in the New World.

## ﻿Materials and methods

Kawakita and Kato (2009: fig. 3) listed six independent origins of obligate moth pollination and these groups will be treated here (sequence as in Kawakita and Kato (2009)). For each group, simple qualitative descriptions or comparisons are provided of the general type of staminate and pistillate flowers. The species selected are those mentioned either by Kawakita and Kato (2009) or by Bouman et al. (2021) in the new phylogeny of the Phyllantheae. Qualitative descriptions of staminate and/or pistillate flowers were made if specimens were available in the herbarium material of Naturalis Biodiversity Center (L, U and WAG in Index Herbariorum, http://sweetgum.nybg.org/science/ih/), but for many species, specimens were lacking or they were too poor to be used or they only showed one sex. The flowers in the moth-pollinated taxa (red-boxed in Fig. 1) are compared with taxa in their sister group (green-boxed in Fig. 1) to observe possible morphological changes in flowers, likely induced by the moth pollination. The groups treated here are all Old World taxa. In a more recent paper, Kawakita et al. (2019) also demonstrated moth pollination in the New World (Fig. 1B), so these taxa, blue-boxed in Fig. 1, are discussed as well.

The genus *Phyllanthus* in its present circumscription is paraphyletic because *Breynia* (including *Sauropus*), *Glochidion* and *Synostemon* are part of it. Two views exist, either to include the four genera into one large genus (e.g. Hoffmann et al. (2006)) or to subdivide *Phyllanthus* in smaller, recognisable genera (e.g. van Welzen et al. (2014)). The latter option has now become feasible with a phylogeny based on a much larger sampling (Bouman et al. 2021). In the rest of the text, we will present the current names and the new classification of Bouman et al. (2022) with the newly-proposed names. Fig. 1 shows the new classification and simplified phylogeny of tribe *Phyllantheae* (Bouman et al. 2022). Table 1 shows the names in *Phyllanthus* as used by Kawakita and Kato (2009) and the new names according to Bouman et al. (2022).

**Table 1.** Groups and species with obligate pollination mutualism and parasitic pollination in Kawakita and Kato (2009) and Bouman et al. (2022).

Kawakita and Kato (2009)	Bouman et al. (2022)
**Obligate Pollination Mutualism**	
**Goup 1: *Breynia* J.R.Forst. & G.Forst.**	**Breyniasubgen.Breyniasect.Breynia**
*B.disticha* J.R.Forst. & G.Forst.	idem
*B.fruticosa* (L.) Müll.Arg.	idem
*B.olongifolia* (Müll.Arg.) Müll.Arg.	idem
*B.retusa* (Dennst.) Alston	idem
*B.vitis-idaea* (Burm.f.) C.E.C.Fisch.	idem
**Group 2: *Glochidion* J.R.Forst. & G.Forst.**	**Glochidionsubgen.Glochidion**
*G.acuminatum* Müll.Arg.	idem
*G.lanceolatum* Hayata	idem
*G.obovatum* Siebold & Zucc.	idem
*G.rubrum* Blume	idem
*G.zeylanicum* (Gaertn.) A.Juss.	idem
**Group 3: *Phyllanthus* L. (no subgen., Madagascar)**	**CiccaL.subgen.Menarda**
	**(Comm. ex A.Juss.) R.W.Bouman**
*P.humbertii* (Leandri) Petra Hoffm. & McPherson	*C.humbertii* (Leandri) R.W.Bouman
*P.marojejiensis* (Leandri) Petra Hoffm. & McPherson	*C.marojejiensis* (Leandri) R.W.Bouman
**Group 4: *Phyllanthus* L.**	***Dendrophyllanthus* S.Moore**
**subgen. Gomphidium (Baill.) G.L.Webster**	**sect. Dendrophyllanthus**
*P.bourgeoisii* Baill.	*D.bourgeoisii* (Baill.) R.W.Bouman
*P.chamaecerasus* Baill.	*D.chamaecerasus* (Baill.) R.W.Bouman
*P.caudatus* Müll.Arg.	*D.caudatus* (Müll.Arg.) R.W.Bouman
*P.mangenotii* M.Schmid	*D.mangenotii* (M.Schmid) R.W.Bouman
**Group 5: *Phyllanthus* L.**	***Dendrophyllanthus* S.Moore**
**subgen. Gomphidium (Baill.) G.L.Webster**	**sect. Leptonema (Baill.) R.W.Bouman**
*P.aeneus* Baill.	*D.aeneus* (Baill.) R.W.Bouman
*P.gneissicus* S.Moore	*D.gneissicus* (S.Moore) R.W.Bouman
*P.guillauminii* Däniker	*D.guillauminii* (Däniker) R.W.Bouman
*P.vulcani* Guillaumin	*D.vulani* (Guillaumin) R.W.Bouman
**Group 6: *Phyllanthus* L.**	**KirganeliaA.Juss.sect.Kirganilia**
**subgen. Kirganelia (A.Juss.) Kurz**	
*P.reticulatus* Poir.	*K.reticulata* (Poir.) Baill.
*P.* sp.	*K.* sp.
**Parasitic pollination**	
**PhyllanthusL.subgen.Swartziani**	***Moeroris* Raf.**
**(G.L.Webster) Ralim. & Petra Hoffm.**	**subgen. Swartziani (G.L.Webster) R.W.Bouman**
*P.amarus* Schumach. & Thonn.	*M.amara* (Schumach. & Thonn.) R.W.Bouman
	***Emblica* Gaertn.**
*P.lepidocarpus* Siebold & Zucc. (= *P.urinaria* L.)	*E.urinaria* (L.) R.W.Bouman
**PhylanthusL.subgen.Isocladus G.L.Webster**	**CathetusLour.subgen.Macraea (Wight) R.W.Bouman**
**(must be subgen. Macraea (Wight) Jean F.Brunel)**	
*P.ussuriensis* Rupr. & Maxim.	*C.ussuriensis* (Rupr. & Maxim.) R.W.Bouman

**Figure 2.**
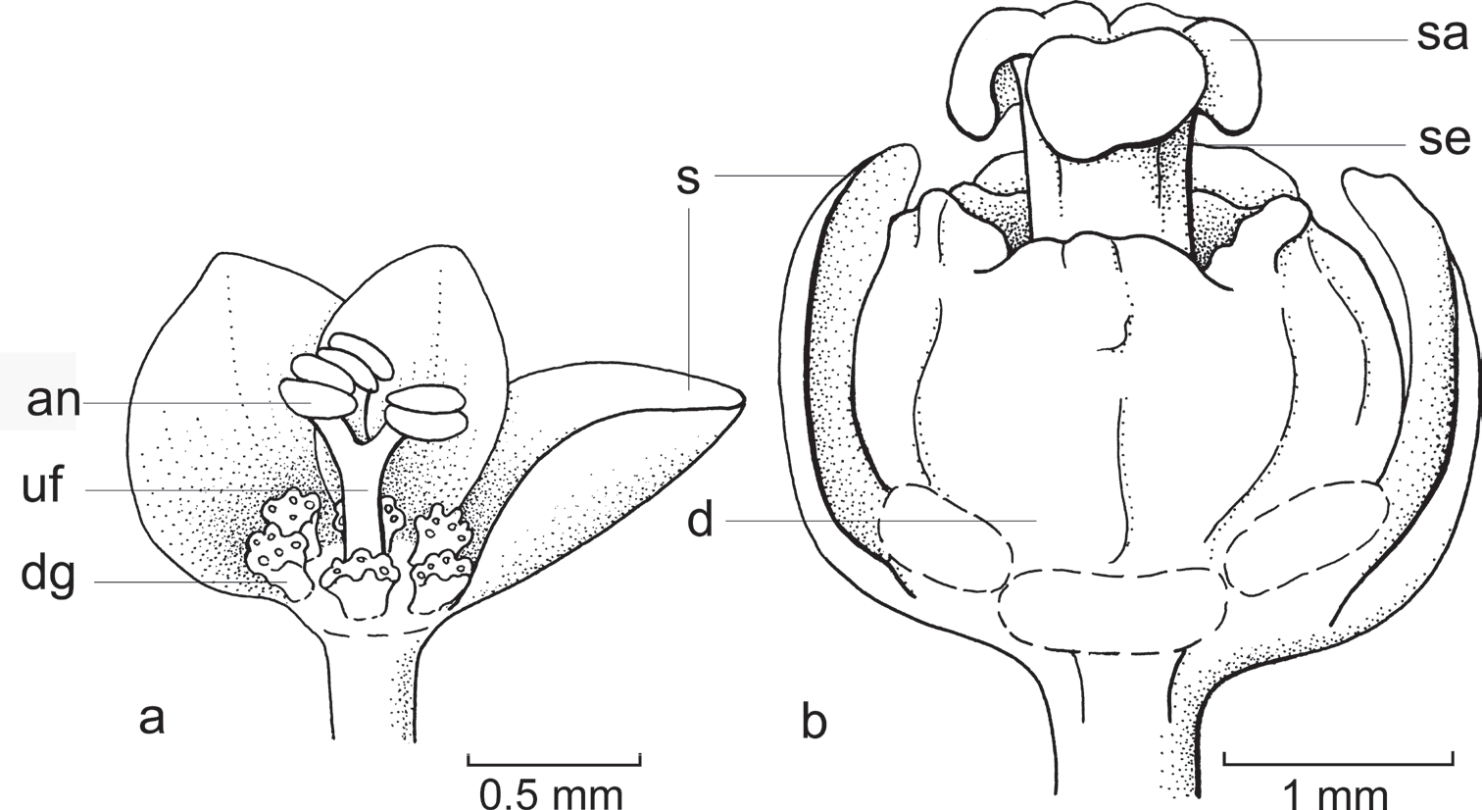
General flower characters of Phyllantheae**a** staminate flower of *Moerorisdebilis* (Klein ex Willd.) R.W.Bouman (formerly *Phyllanthusdebilis* Klein ex Willd.) (re-used with permission, Flora of Thailand 8, 2: fig. 54B2. 2007) **b** pistillate flower of *Cathetusgracilis* (Hassk.) R.W.Bouman (formerly *P.albidiscus* (Ridl.) Airy Shaw); part of sepals of both flowers removed (re-used with permission, Flora of Thailand 8, 2: fig. 55A2. 2007); an = anther; d = disc; dg = disc glands; s = sepals, sa = stigma; se = style; uf = united filaments (**a***Maxwell 97-915***b***Shimizu et al. T-26280*; both in L). Illustrations by Anita Walsmit Sachs, 2007.

**Figure 3.**
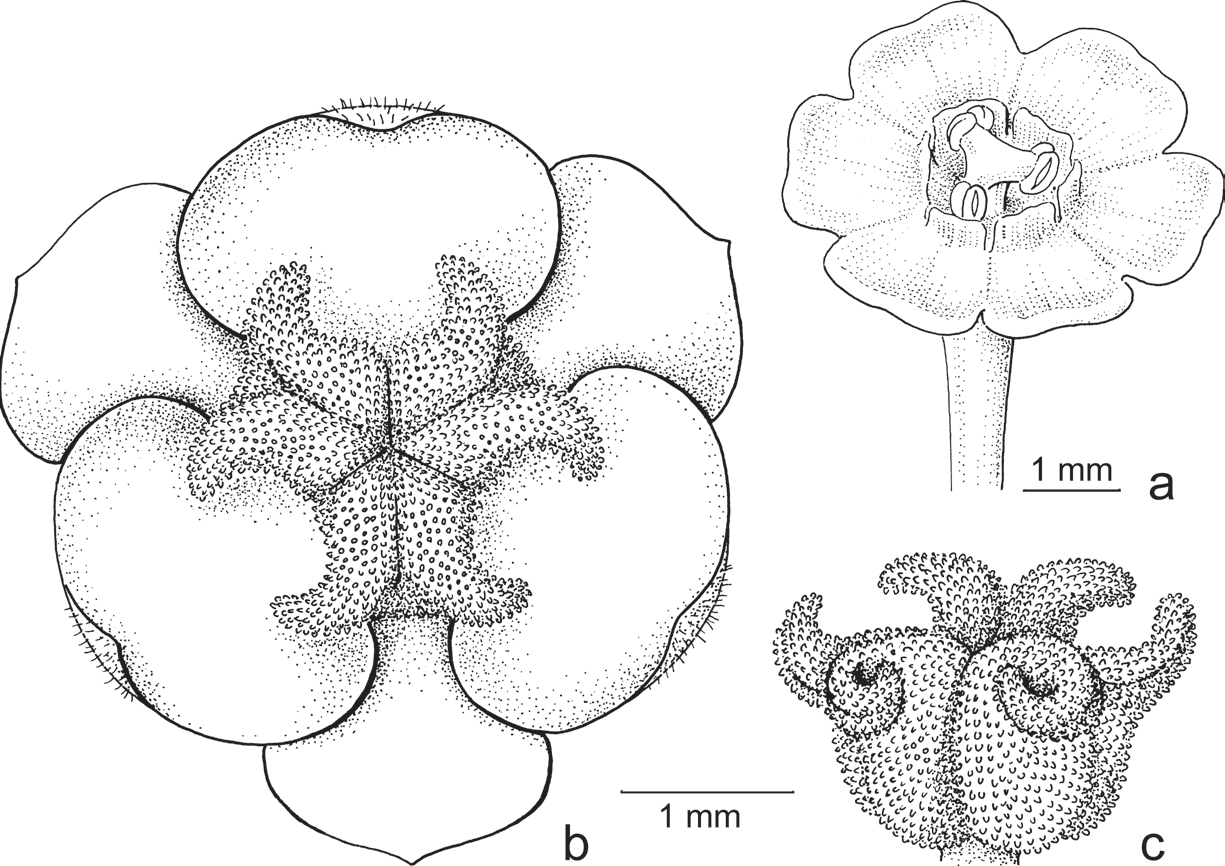
*Breynia* J.R.Forst. & G.Forst. non-moth-pollinated flowers **a** staminate flower of *B.bicolor* (Craib) Chakrab. & N.P.Balakr. (subgen. Breyniasect.Cryptogynium) with clear scales and androecium (re-used with permission, Flora of Thailand 8, 2: fig. 72F. 2007). – *B.lithophila* Welzen & Pruesapan (subgen. Sauropus) **b** pistillate flower **c** gynoecium with papillae (re-used with permission: Thai Forest Bulletin (Botany) 38: 116, fig. 3. 2010) (**a***Maxwell 96-712***b***Phonsena*, *Chusithong*, *de Wilde & Duyfjes 5594*; both in L). Illustrations by: **a** Jan van Os, 2002 **b, c** Anita Walsmit Sachs, 2009.

The terms style and stigma need some explanation as these are sometimes used differently. The style is the united part (of the stigmas) on top of the ovary, it can be present (Fig. 2b) or absent (Fig. 3c). The stigmas are the split part. There are usually three stigmas, which, at the end, usually split into two short lobes. The stigmatic tissue, the part receiving the pollen, is often the papillate part on the adaxial side of the stigmas (Figs 3c, 12b). However, in various species, these are absent (e.g. Fig. 14b) and, therefore, the three arms are considered as stigmas. This is concordant with the use of the terms in the Flora Malesiana revisions of the Euphorbiaceae s.l. (including Phyllanthaceae): www.nationaalherbarium.nl/euphorbs/.

## ﻿Results

The general flower type in the Phyllantheae shows usually six sepals (in two whorls of three), a nectar disc (annular or consisting of three or six separate glands) and then either three or more stamens in the staminate flowers (free or variously connate; Fig. 2a) or an ovary with usually three locules with two ovules per locule and on top a short style branching into the stigmas (Fig. 2b). The latter are often split at the top (Fig. 5c). For each group, the possible changes in these organs are discussed and Table 2 shows short descriptions of both sexes of flowers for the species mentioned in the text and an indication whether or not they are (likely) moth-pollinated.

**Table 2.** Possible adaptations or lack of adaptations to moth pollination in staminate and pistillate flowers. Group refers to the groups as discussed in the text and in Fig. 1, I means Ingroup, O = local outgroup (related clade/taxon with which the ingroup is compared). The classification follows Bouman et al. (2022). Adapted: – = primitively not adapted to moth pollination; + = adapted to moth pollination, obs.+ = moth adaptation observed (Kawakita et al. 2019), but morphologically not adapted (morph.-); R = reversal to non-moth pollination.

Genus/Species	Infrageneric taxon	Group	Staminate flowers	Pistillate flowers	Adapted
***Breynia***	section Breynia	1-I	Tight, upright united sepals, with subapically scales closing flower when young; filaments upright, united, glands along androphore.	Sepals rather small or accrescent; disc and scales absent; stigmas reduced in length.	+
*B.retusa*	section Breynia	1-I	Idem, but flowers more open.	Idem; sepals not accrescent, stigmas well-developed, recurved.	R
*Breynia*	section Cryptogynium	1-O	Sepals partly to complete united, disc-like, scales present (idem); filaments united, splitting apically with anthers underneath (exceptions exist)	Sepals partly united, disc-like; scales absent; stigmas well-developed, separate, flat to ovary, apically split and recurved (exceptions exist)	–
	+ subgen. Sauropus	1-O			
***Glochidion***	subgen. Glochidion	2-I	Tight, upright sepals; no disc; stamens tightly upright, anthers along filament.	Sepals more or less upright; disc absent; stigmas united in cone.	+
		-			
*G.sericeum*	subgen. Glochidion	2I	idem.	2 small sepals; disc absent; stigmas well-developed, reflexed.	R
*Glochidion*	subgen. Phyllanthodendron	2-O	Sepals upright, more loose, long apex; large petal-like	Sepals more loose; disc glands present;	–
	+ subgen. Pseudoactephila	2-O	disc glands; filaments basally united, apically diverging, anthers separate.	stigmas well developed, free, reflecting.	
***Cicca*** *humbertii*	subgen. Menarda	3-I	Sepals upright, narrow inside, small disc glands, stamens vertically united, androphore sturdy, anthers vertically along it.	Sepals tight, no disc observed; stigmas forming cone with small opening.	+
*C.perrieri*		3-I	Sepals stiff, upright, disc glands small; stamens free.	Sepals tight, disc glands; stigmas forming cone with small opening.	+
*C.sambiranensis*		3-I	Sepals upright, not so stiff; disc glands; stamens tight when young, older free	Sepals stiff, thick, upright; disc glands; stigmas forming short cone	+
*C.coodei*		3-O	Open flowers; disc glands present, stamens free, diverging	Disc present; ovary with upright short style and three well-developed stigmas, split till halfway	–
*C.cryptophila*		3-O	Open flowers, disc glands present, stamens free	Disc present, ovary on short gynophore, upright short style, three well-developed spreading stigmas, split	–
*C.betsilaeana*	subgen. Betsileani	3-O	Sepals thin; disc glands; stamens free.	Sepals thin; disc circular; stigmas well-developed, spreading, largely apically split.	–
**Dendrophyllanthussect.Dendrophyllanthus**					
*D.aphanostyla*	sect. Dendrophyllanthus	4-I	Not seen	Stigmas very short, cone-like, split, in circle	+
*D.bourgeoisii*	sect. Dendrophyllanthus	4-I	Not seen	Stigmas short, free, apically slightly split, in circle around opening	+
*D.castus*	sect. Dendrophyllanthus	4-I	Not seen	Stigmas upright, free, forming circle around opening	+
*D.clamboides*	sect. Dendrophyllanthus	4-I	sepals 6, ± equal, disc lobes 6, thick; stamens 3, united.	Sepals 6, large, inner broader; disc lobes 6, ovary 3-locular, on top style and three, upright, split stigmas in tight circle.	+
*D.cuscutiflorus*	sect. Dendrophyllanthus	4-I	Not seen	stigmas upright in small cone, apices split.	+
*D.dzumacensis*	sect. Dendrophyllanthus	4-I	Not seen	Stigmas short, free, forming cone, tips slightly bifid.	+
*D.effusus*	sect. Dendrophyllanthus	4-I	Sepals 6, big 2-lobed disc glands, three united stamens, stout androphore, 3 anthers upright, united.	Sepals 6, outer three smaller, disc ring-like, margin erose, ovary 3-locular, style long, stigmas, upright, in circle with opening in middle.	+
*D.glochidioides*	sect. Dendrophyllanthus	4-I	(bud) sepals 6, inner much larger, six disc glands, stamens 3, filaments united, apically split and diagonally upwards, stamens underneath.	Sepals 6, inner larger, six disc glands; ovary 3-locular, style long with on top united stigmas, upright, forming tight circle.	+
*D.kostermansii*	sect. Dendrophyllanthus	4-I	Three outer small sepals, three inner large, closing flower. Six disc glands, stamens erect, free, filaments with anther 3-locular, stigmas erect, forming circle, slightly along at abaxial side, connective appendiculate.	(young) Sepals 6 in two whorls, disc circular, ovary bilobed at apex.	+
*D.mangenotii*	sect. Dendrophyllanthus	4-I	Not seen	Stigmas free, narrow, upright, apically not split.	+
D.poumensisvar.poumensis	sect. Dendrophyllanthus	4-I	Not seen	Stigmas forming connected high dome	+
*D.rosselensis*	sect. Dendrophyllanthus	4-I	(bud) sepals 6, outer narrower, Three large disc glands; stamens 3, filaments likely united.	Sepals 6, inner larger, disc ring-like; ovary 3-locular, apically with ring of erect stigmas, apically split.	+
*D.buxoides*	sect. Dendrophyllanthus	4-O	Not seen	Style sturdy, three well-developed, broad stigmas upright but slightly diverging	+/–?
*D.caudatus*	sect. Dendrophyllanthus	4-?	Not seen	Style stout, stigmas 3, long, free, very slender, apically not split, slightly curled inwards	+/–?
*D.finschii*	sect. Dendrophyllanthus	4-O	Not seen	Stigmas free, well-developed, apically split.	–
D.poumensisvar.longistylis	sect. Dendrophyllanthus	4-O	Not seen	Stigmas separate	–
*D.pancherianus*	sect. Dendrophyllanthus	4-O	Not seen	Stigmas well-developed, bent backwards.	–
*D.tabularis*	sect. Dendrophyllanthus	4-O	Sepals 6, inner larger, three large disc glands, stamens 3, large, filaments united, anthers apiculate and large.	Sepals 6, inner larger, disc?, ovary 3-locular, stigmas well-developed, recurved, apically split	–
*D.tenuirhachis*	sect. Dendrophyllanthus	4-O	Not seen	Stigmas upright, well-developed, apically split	–
*D.wilkesianus*	sect. Dendrophyllanthus	4-O	Sepals 6, inner broader; disc gland 3, large; stamens 3, free, anthers short.	(young fruit) sepals 6, inner broader, disc glands 6, small, ovary 3-locular, stigmas well-developed	–
**Dendrophyllanthussect.Leptonema**					
*D.aeneus*	sect. Leptonema	5-I	Not seen	Sepals 5, circular disc, ovary 3-locular, on top columnar stigma of which some spreading	+
*D.ligustrifolius*	sect. Leptonema	5-I	sepals 5, disc glands 5, stamens 3, free.	(young fruit): 3-locular, on top columnar style and stigma, apex not split; in fruit completely splitting.	+
*D.favieri*	sect. Leptonema	5-?	Sepals fleshy, 5, inner larger; disc ring-like, stamens 5, two free outer, three partly united inner.	Not seen	?
*D.bupleuroides*	sect. Leptonema	5-O	Not seen	Stigmas well-developed, spreading, not apically split	–
*D.hypospodius*	sect. Leptonema	5-O	Sepals 5, disc glands 5, free stamens, three inner longer than two outer.	Style short, stigmas well-formed, spreading, apically very slightly split/erose.	–
*D.kanalensis*	sect. Leptonema	5-O	Sepals 5, outer two smaller, bilobed disc glands; stamens five, filaments united, two outer, three inner.	Sepals 5, outer two smaller, disc narrow ring; ovary 3-locular, apically short spreading stigmas.	–
*D.lacunarius*	sect. Leptonema	5-O	Sepals 6, disc glands, 3 stamens, mainly free, upright.	Style short, stigmas well-developed, spreading, apically split, completely splitting in fruit.	–
*D.loranthoides*	sect. Leptonema	5-O	Not seen	Sepals 5/6, small, inner larger; disc glands large, 2-lobed; ovary 3-locular, spreading, sessile, well-developed stigmas, apically not split.	–
*D.sauropodoides*	sect. Leptonema	5-O	Sepals 5, disc glands five with kind of honey-comb structure on top, stamens 5, free, three central, all upright.	Style present; stigmas 3, well-developed, spreading, apically bifid.	–
*D.serpentinus*	sect. Leptonema	5-O	Disc ring-like; stamens 5, free, two outer, three inner.	Style short, stigmas well-developed, spreading, apically (seemingly) not split.	–
*D.vulcani*	sect. Leptonema	5-O	Sepals 5, outer two smaller, many disc glands, also inside stamen ring; stamens 5 in one whorl.	Sepals small, thick; disc inconspicuous; ovary 3-locular, stigmas 3, widely spreading, sessile, very slightly splitting apically.	–
***Kirganelia*** *reticulata*	section Kirganelia	6-I	Small, sepals thin, small disc glands, stamens in two whorls, outer free, inner filaments basally united	Small, sepals thin, small disc glands, ovary on top four or five short, apically slightly split stigmas, bent towards each other and forming a cone.	+
*K.somalensis*	section Pseudomenarda	6-O	Larger than former, five sepals, five disc lobes, five stamensin two whorls, outer two free, inner basally united.	Sepals 5, relatively large, open, ring disc, 5-locular ovary with short style and five horizontal well-developed stigmas apically split to 1/3	–
**New World Herbaceous species**					
*Moerorisamara*	subgenus Swartziani	-	Small, hanging down, open	Small, hanging down; stigmas short, well-developed, spreading.	–
*Moerorisstipulata*	subgenus Moeroris	-	Small, hanging down, open	Small, hanging down; stigmas short, well-developed, spreading.	–
*Phyllanthusorbiculatus*	sbg. *Conami* sct. *Apolepis*	-	Small, hanging down, open	Small, hanging down; stigmas short, well-developed, spreading.	–
**PhyllanthussubgenusCiccastrum**					
*P.purpusii*	subgenus Ciccastrum	O	six sepals, outer three smaller, inner stiff and upright, forming narrow cylinder, disc glands, three united stamens with apically anthers along it.	Sepals 6, like staminate, ring-like disc; 3-locular ovary, short style, 3 spreading stigmas, recurved, apically split.	♂+ ♀–
*P.riedelianus*	subgenus Ciccasrum	O	Sepals 6, disc glands 6, stamens 3, upright, partly united, anthers free, slightly spreading.	Young fruit: sepals and disc glands caducous; ovary 3-locular, with three sessile free stigmas, bent upwards and towards each other (only fruit?)	–
**PhyllanhussubgenusConami & *Xylophylla***					
	subgenus Conami				
*P.acuminatus*	section Conami	7-O	Open, anthers pointing downward from filaments.	Open, stigmas well-developed, spreading over ovary, tips broadened and slightly split.	–
*P.graveolens*	section Conami	7-I	Open, stamens free, obliquely upright.	Open, stigmas short, well-developed and spreading over ovary	obs.+ morph.±
	subgenus Xylophylla				
*P.huallagensis*	section Elutanthos	7-I	In bud, sepals soft, stamens united, apically splitting anthers underneath.	sepals soft, stigmas well-developed, radiating horizontally.	obs.+ morp.–
*P.salviifolius*	section Oxalistylis	7-I	Open, soft sepals, stamens with horizontally bent anthers (opening downward)	Open, soft sepals, three stigmas spreading apices fan-like.	obs.+ morph.–
**PhyllanthussubgenusMicroglochidion**					
*P.duidae*		8-I	Sepals 6, disc glands 6; stamens 3, free, large, erect.	Sepals 6; disc ring; gynophore short; ovary 3-locular, stigmas 3, united, at top 3-lobed	+
*P.majus*		8-I	not seen	Young: sepals 6; disc ring; ovary 3-locular, stigmas united (as thick as ovary), upright, cone-like, top-3 lobe, bent inside, each lobe bifid	+
*P.pycnophyllus*		8-I	Sepals 6, three large disc glands, three free large stamens.	Young: six sepals, disc likely present; ovary 3-locular, stigmas upright, grown together, small teeth around opening apically.	+
*P.vacciniifolius*		8-I	Sepals 6, inner larger; disc glands 3; stamens 3, basally united and attached to inner sepals, upright, most part free, connectives extended.	Pedicel strongly thickened apically and invaginated under sepals; sepals 6, small disc; ovary 3-locular, stigmas united, upright, 3-lobed at top, not split.	+
*P.lediformis*		8-O	Young; sepals 6; disc glands 3; stamens 3, free, erect, anthers long, seems that connective appendage is developing	Sepals 6; disc not seen, too poor material; ovary 3-locular, apically style splitting into three flat stigmas, broadened at apex.	–
*P.maguirei*		8-O	Sepals 6, spreading, top inrolled; disc glands 6; stamens 3, free, anthers on top bent downwards.	Not seen	–
P.myrsinitesssp.myrsinites			Sepals 6, weak, spreading; disc glands 6, stamens 3, free, on slightly higher receptacle.	Sepals 6, disc ring-like; ovary 3-locular, stigmas 3, well-developed, free, recurved over ovary, tips split.	–
*P.neblinae*			not seen	Older fruit: stigmas 3, well-developed, spreading, flat, apically split and divergent (like crescent-moon)	–
**PhyllanthussubgenusXylophyllasectionEpistylium**					
*P.axillaris*	section Epistylium	n.a.-I	Buds too young	In bud: stigmas forming closed cone	+

### ﻿*Breynia* versus former *Sauropus* (Group 1 in Fig. 1A; Table 2)

Formerly, *Sauropus* (van Welzen, 2003) was a separate genus of which the species are not pollinated by moths. Based on phylogenetic analyses (Pruesapan et al. 2008, 2012) both genera had to be united under the oldest name *Breynia* (van Welzen et al. 2014). Former *Sauropus* is now divided over Breyniasubgen.Breyniasect.Cryptogynium Welzen & Pruesapan and Breyniasubgen.Sauropus Welzen & Pruespan (Fig. 1A).

Both genera were formerly separated because of the strong differences in floral morphology in both sexes. The flowers of both sexes in former *Sauropus* are usually flat, open and disc-like. The staminate flowers (Fig. 3a) have six sepals, basally to almost completely united; they lack nectar glands, but (usually) have scales inside, which are probably transformed disc glands. The scales keep the flower closed when the stamens are still immature. The filaments of the three stamens are basally united into a column, but at the top they form three, ± horizontal, free arms with the anthers underneath. The ovary of the pistillate flowers (Fig. 3b) has a flat top, on which there are three, well-developed stigmas that are usually flat and split at the apex and resemble a crescent-moon with papillae to catch pollen (Fig. 3c). Exceptions are that, in some species, the stigmas are erect and, in some, the staminate flowers lack the scales, but instead they have three sepals that are folded inwards and the stamens are enlarged and point diagonally upwards. The potential pollinators of these ‘*Sauropus*-flower’ species have not yet been elucidated by any study.

**Figure 4.**
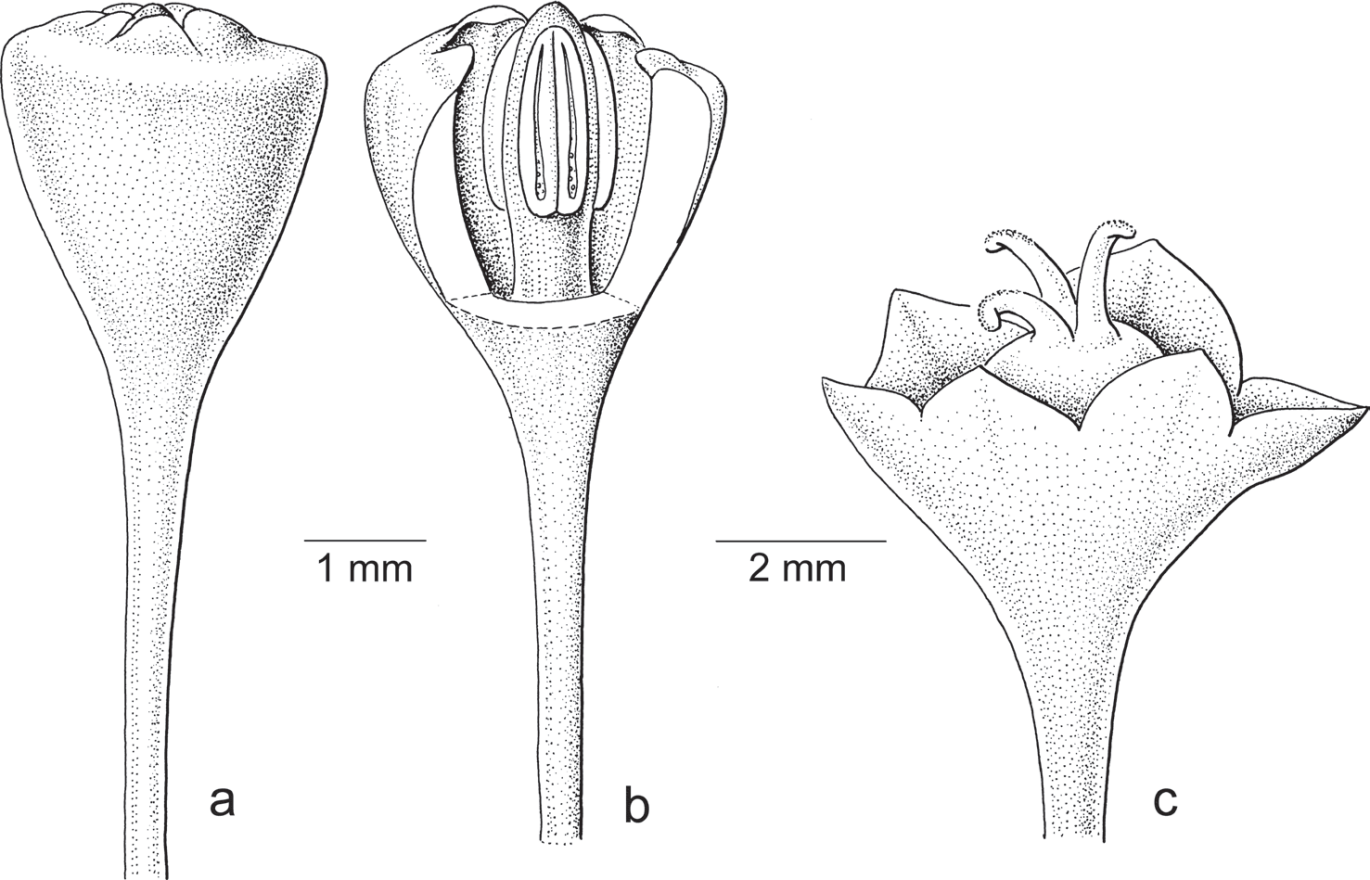
*Breyniaglauca* Craib **a** staminate flower with scales slightly upright, calyx lobes reduced to thickened ring **b** idem with part of calyx removed, showing androecium **c** pistillate flower with three entire stigmas, style absent. Illustrations by Jan van Os, 2002. Re-used with permission of the Flora of Thailand (Flora of Thailand 8, 1: fig. 30B–D. 2005).

Breyniasubgen.Breyniasect.Breynia (Fig. 1A) is pollinated by moths (Kawakita and Kato 2004b, 2009). The staminate flowers are narrow and campanulate, with united sepals forming a tube, with the disc scales on the top inside (sometimes the sepal lobes are reduced to a thickened ring and the scales resemble the calyx lobes; Fig. 4a). Within the tube, three stamens are united into a massive stalk (androphore) with the anthers orientated vertically along it (Fig. 4b). The pistillate flowers are less open than in former *Sauropus*, but the main difference is in the stigmas. The style is lacking and the stigmas are greatly reduced in size, sometimes completely split (Fig. 4c). In some species, there are six short vertical stalks on top of the ovary without any stigmatic papillae. Only two species in this section, *B.fruticosa* (L.) Müll.Arg. and *B.glauca* Craib (Fig. 4), still have larger stigmas, but these are not united in a style.

Furukawa and Kawakita (2017) showed that a gynophore (of variable length) often develops after fruit set in *B.officinalis* Hemsl. on Amami-Oshima Island (Ryukyu Islands, Japan), also mentioned by Zhang et al. (2012), which likely precludes overconsumption by *Epicephalavitisidaea* Li, Wang & Zhang, 2012, as the larvae have to eat their way up through the gynophore (unfortunately, they used the name *B.vitis-idaea* (Burm.f.) C.E.C.Fisch., a species not present in the Ryukyu Islands and southern China and which definitely lacks a gynophore; see van Welzen and Esser (2005)).

**Figure 5.**
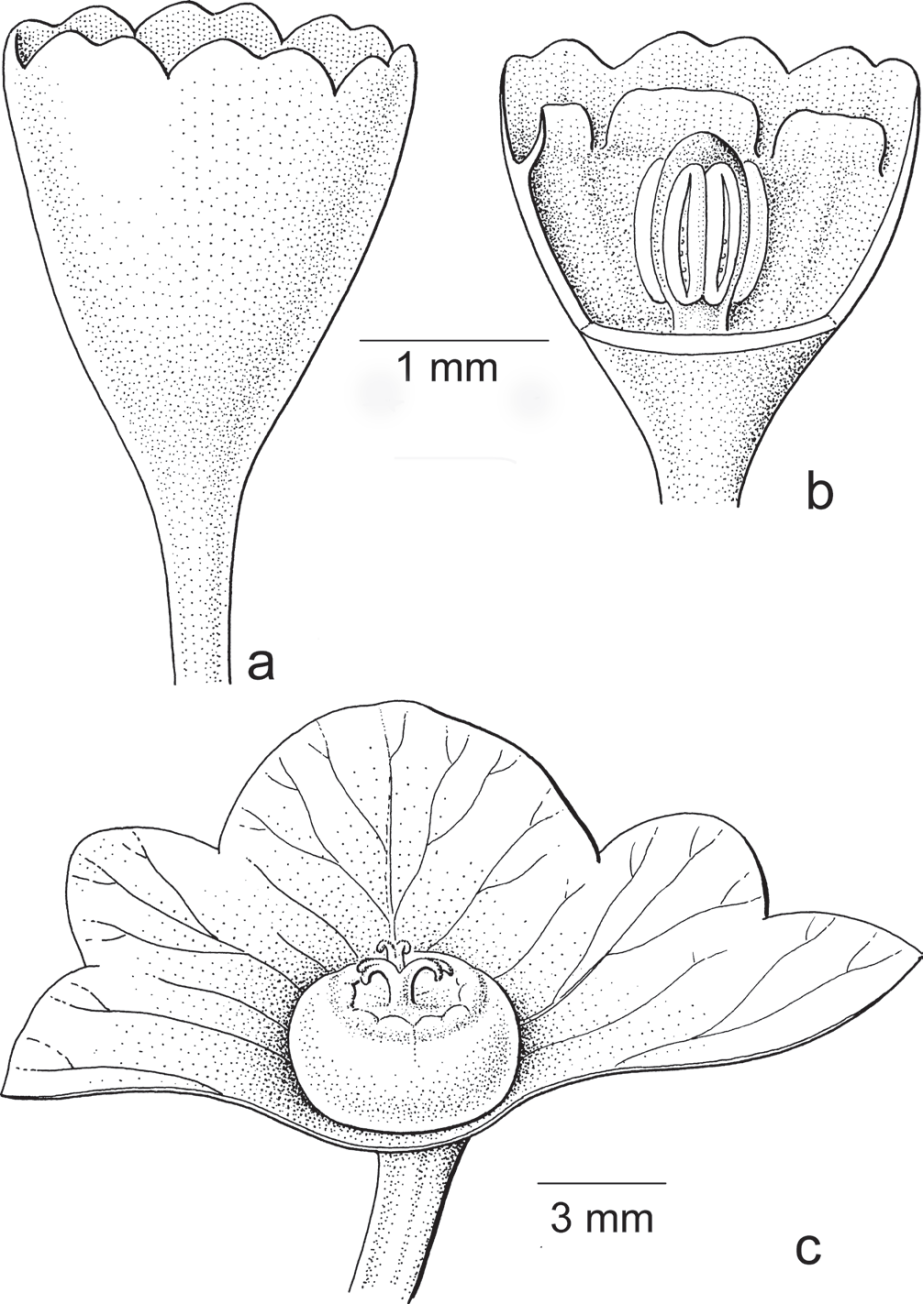
*Breyniaretusa* (Dennst.) Alston **a** staminate flower **b** idem, part of calyx removed, androecium and scales visible **c** pistillate flower with part of calyx removed, distinct style and branching stigmas visible. Illustrations by Jan van Os, 2002. Re-used with permission of the Flora of Thailand (Flora of Thailand 8, 1: fig. 29A, B, D. 2005).

Seemingly, in comparison to the outgroup, the flowers in the ancestral species of sect. Breynia evolved in response to moth pollination: the staminate flowers became closed, campanulate and, inside, the anthers became vertical with united stamens (Fig. 4a, b); in the pistillate flowers, the stigmas are strongly reduced (Fig. 4c). *Breyniaretusa* shows a reversal towards a normal style and stigma (Fig. 5c), thus likely this species underwent a host switch. Kato and Kawakita (2017: table 6.1) recorded that *B.retusa* is not pollinated by *Epicephala* moths, but the (new) pollinators are unknown. The staminate flowers of *B.retusa* are rather narrow (Figs 5a, b), but more open than other flowers of the moth-pollinated species.

Stigmatic papillae are often present in Breyniasubgen.Breyniasect.Cryptogynium and Breyniasubgen.Sauropus (Fig. 2c), but absent or strongly reduced in Breyniasubgen.Breyniasect.Breynia (Fig. 4). The disappearance of the papillae may be another adaptation to moth pollination.

### ﻿*Glochidion* versus former PhyllanthussubgenusPhyllanthodendron (Group 2 in Fig. 1A; Table 2)

**Figure 6.**
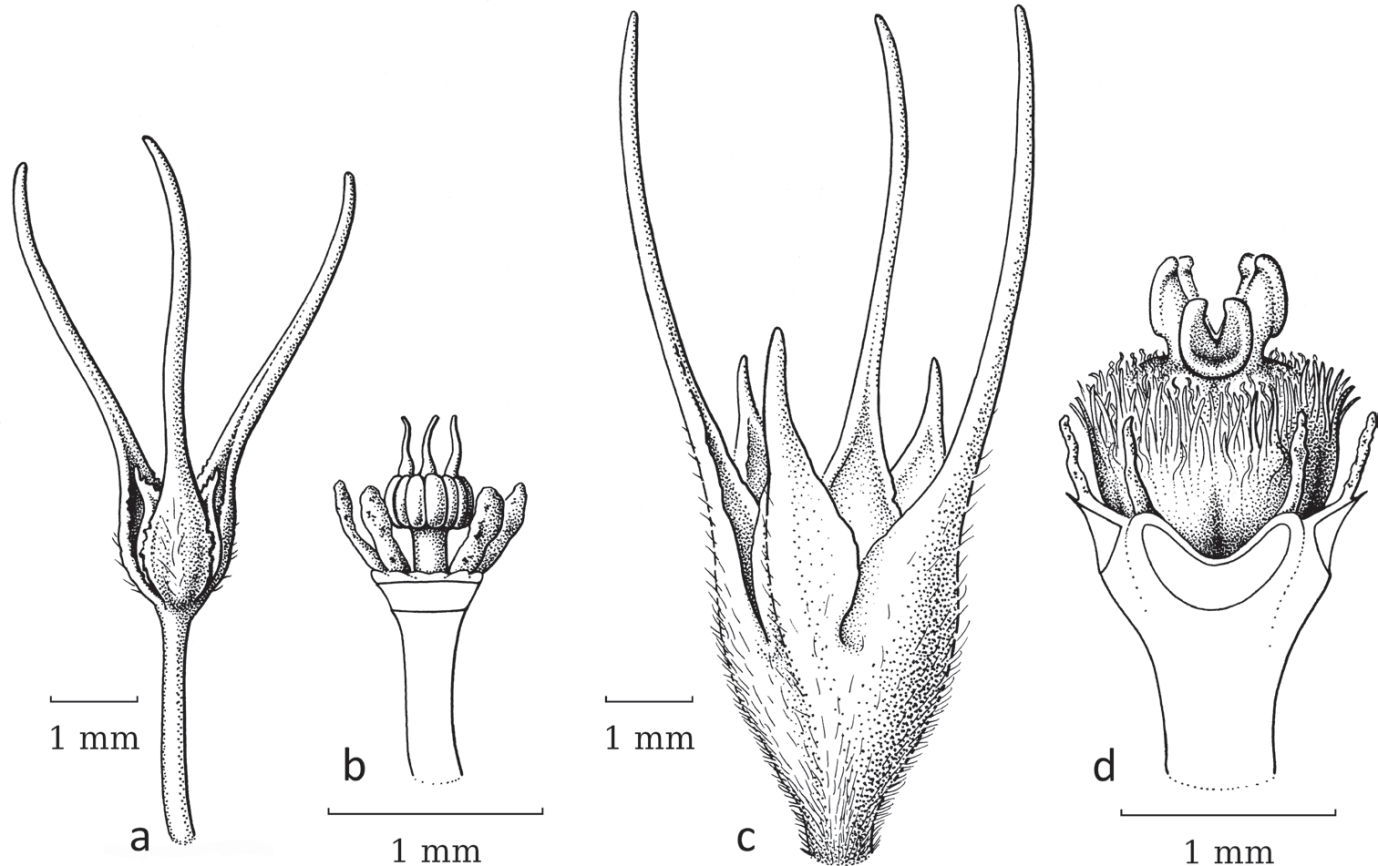
*Glochidionminutiflorum* (Ridl.) R.W.Bouman (formerly Phyllanthus (subg. Phyllanthodendron) ridleyanus Airy Shaw **a** staminate flower which has three outer sepals with elongated apices **b** idem, part of sepals removed, petaloid disc glands visible and stamens with appendices on the connectives **c** pistillate flower **d** pistillate flower with part of sepals removed, petaloid disc glands visible and hairy ovary with the stigmas on top (**a, b***Kiew & Anthonysamy 2977***c, d***Stone 9494*; both L). Illustrations by Esmée Winkel, 2021.

A similar situation as with *Breynia* occurred in these two groups. Former *Phyllanthodendron* Hemsl. (or PhyllanthussubgenusPhyllanthodendron (Hemsl.) G.L.Webster) is presently split into Glochidionsubgen.Phyllanthodendron (Hemsl.) R.W.Bouman and G.subgen.Pseudoactephila (Croizat) R.W.Bouman. These two taxa are not moth-pollinated (Fig. 1A; Kato and Kawakita (2017)). The staminate flowers of former *Phyllanthodendron* are rather open, with five or six basally connate sepals with an attenuate apex (Fig. 6a, b), the disc glands are often large and somewhat petal-like, the generally three stamens have connate filaments and diverging upper parts and the connectives have an apical appendage. The pistillate flowers (Fig. 6c, d) have similar sepals and disc glands as the staminate flowers and on top of the ovary are well-developed stigmas (often with a short style) that are apically entire or shortly bifid.

**Figure 7.**
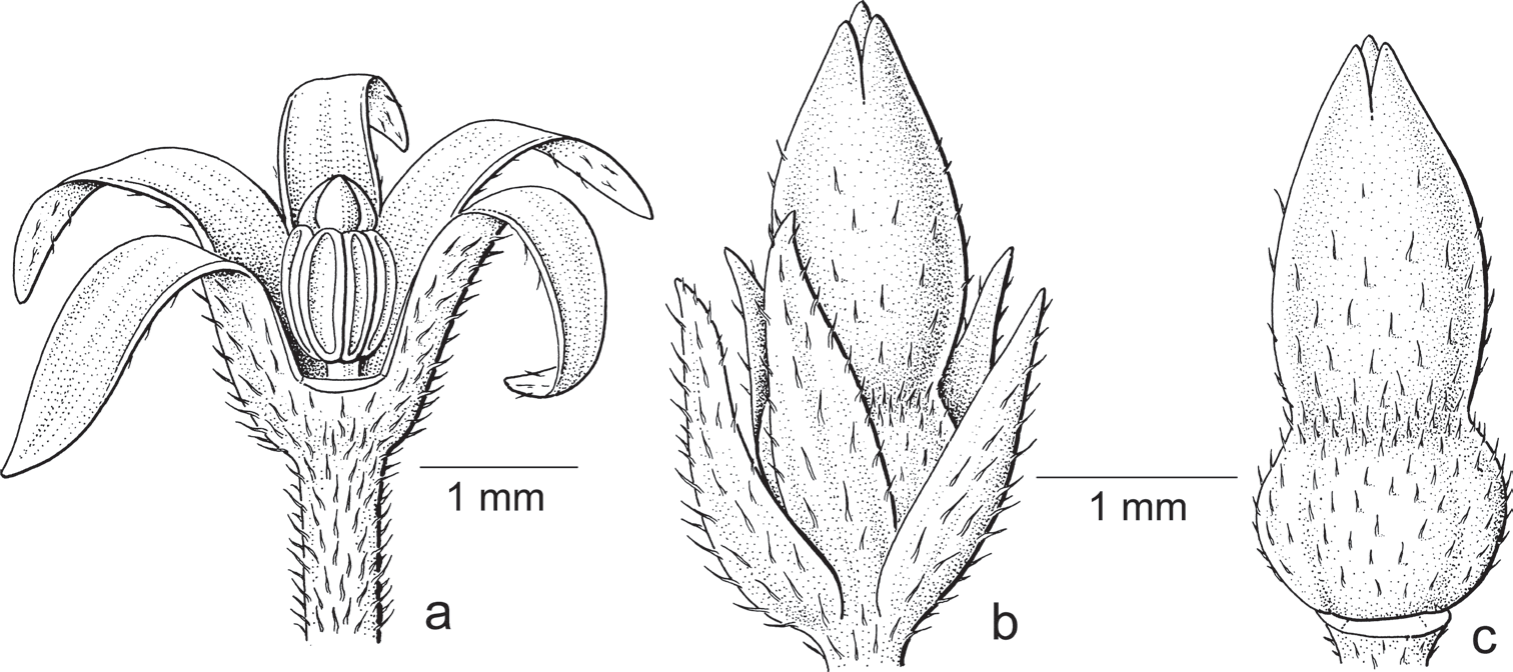
*Glochidionrubrum* Blume **a** staminate flower with sepal removed, androecium of adnate stamens with apically appendaged connectives **b** pistillate flower **c** gynoecium with upright, united stigmas with central cavity. Drawing: Jan van Os, 2003. Re-used with permission of the Flora of Thailand (Flora of Thailand 8, 2: fig. 5C–E. 2007).

In subgenus Glochidion, which is moth-pollinated, flowers of both sexes lack a disc. The staminate flowers (Fig. 7a) are campanulate and the androecium resembles that of *Breynia* (Fig. 5b) as the filaments are vertical and tightly together with the anthers along them and the connectives have apical appendages that form a pyramidal cone. When wilting, the stamens start to detach from each other and bend outwards and then resemble the stamens of the staminate flowers in *Glochidion* subgenera *Phyllanthodendron* and *Pseudoactephila*. In the pistillate flowers (Fig. 7b, c), the stigmas are upright and united into a cone terminating in the often slightly split apices of the stigmas that form a cavity in the middle where the female moth deposits the pollen (Fig. 7b, c).

**Figure 8.**
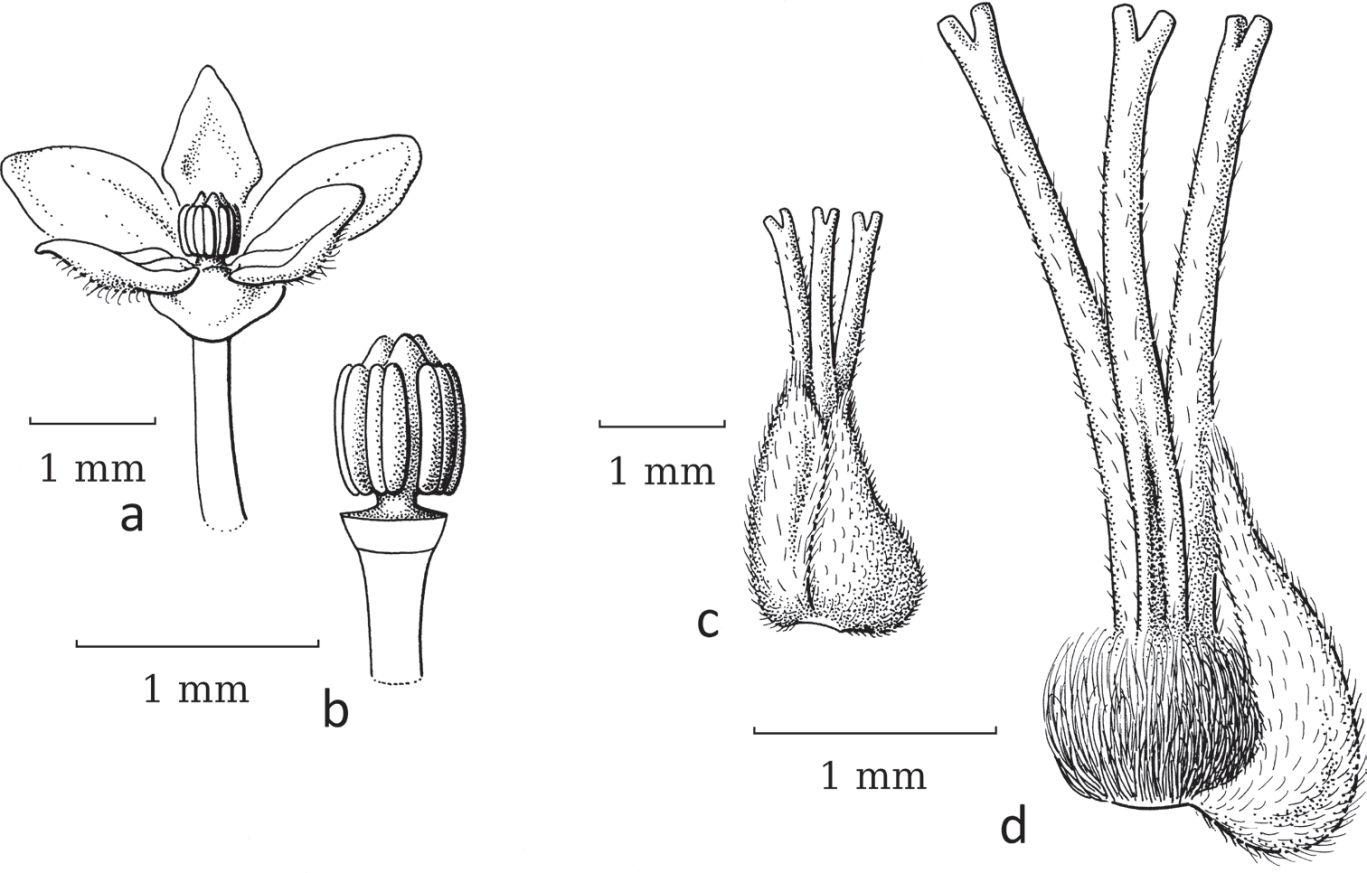
*Glochidionsericeum* (Blume) Zoll. & Moritzi **a** staminate flower **b** staminate flower with part of sepals removed, showing adnate stamens **c** pistillate flower with a reduced number (2) of sepals **d** pistillate flower with sepal removed showing well-developed style and stigmas (*Sinclair 10680*, L). Illustration by Esmée Winkel, 2021.

As with the first group (*Breynia*, including former *Sauropus*), the differences in flower morphology led to this group being separated into two genera. Based on the phylogeny (Bouman et al. 2021) and the resulting new classification (Bouman et al. 202), both former genera are now united under *Glochidion* (*Phyllanthodendron* was paraphyletic and *Glochidion* is the older name). The staminate flowers in subgenus Glochidion (Fig. 1A) are narrower than in the other two subgenera with more strongly united stamens and the connective appendages touching, while in the pistillate flowers, the stigmatic surface is reduced by uniting the stigmas into an erect pyramidal structure. In both sexes, the disc/disc glands are gone, as seemingly the moths do not need a nectar stimulus. As in *Breynia*, there is one *Glochidion* species, *G.sericeum* (Blume) Zoll. & Moritzi, showing a reversal, not in the staminate flowers (Fig. 8a, b) but in the pistillate flowers, where a well-developed style (covered by the sepals) with free, spreading stigmas is present (Fig. 8c, d); Kato and Kawakita (2017: fig. 10.2) did not record any *Epicephala* moth on this species.

### ﻿CiccasubgenusMenarda versus CiccasubgenusBetsileani (Group 3 in Fig. 1; Table 2)

*Cicca* L. is separated from *Phyllanthus* (Bouman et al. 2022) and restricted to a few subgenera, most of which are only found in Madagascar. Of the local outgroup, CiccasubgenusBetsileani (Jean F.Brunel) R.W.Bouman, only *C.betsileani* (Leandri) R.W.Bouman could be studied, a non-moth-pollinated species. Its flowers are open with six thin sepals; the staminate flowers have six disc glands and three free stamens; the pistillate flowers have a 3-locular ovary bearing terminal, well-developed, spreading stigmas that are split in the upper 2/3.

**Figure 9.**
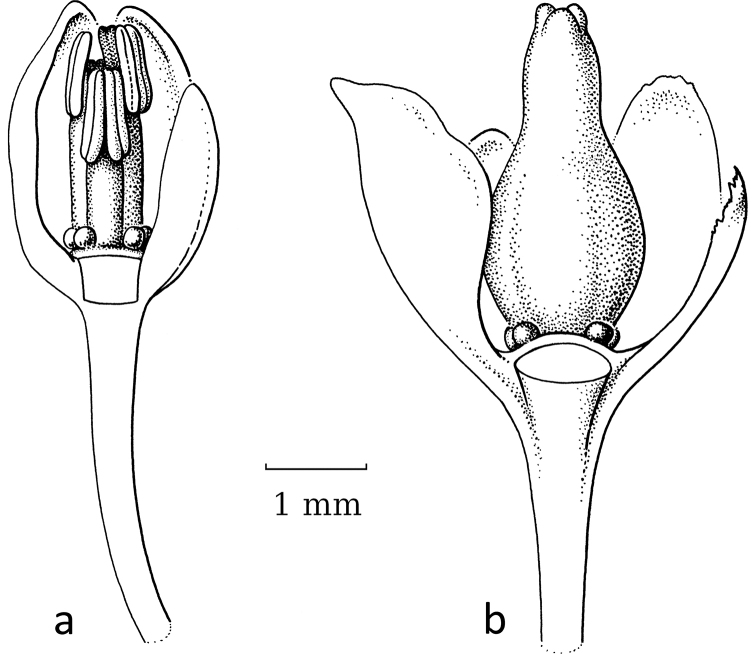
*Ciccaperrieri* (Leandri) R.W.Bouman **a** staminate flower with part of sepals removed showing free, upright stamens **b** pistillate flower with part of sepals removed showing upright, united stigmas (*Gautier LG 3009*, WAG). Illustration by Esmée Winkel, 2021.

CiccasubgenusMenarda (Comm. ex A.Juss.) R.W.Bouman has staminate and pistillate flowers with 5(6) sepals; the staminate flowers have separate disc glands and three or five stamens; the pistillate flowers show a circular disc or separate disc glands and a 3-locular ovary (a revision and drawings can be found in Ralimanana and Cable (2020)). In Kato and Kawakita (2017), subgenus Menarda is represented by *Ciccahumbertii* (Leandri) R.W.Bouman and *C.marojejiensis* (Leandri) R.W.Bouman (latter not included in this study) as being moth-pollinated. Various species of subgenus Menarda were investigated. Morphologically, *Ciccahumbertii* (Leandri) R.W.Bouman, *C.perrieri* (Leandri) R.W.Bouman (Fig. 9) and *C.sambiranensis* (Leandri) R.W.Bouman have staminate flowers with (rather) stiff, upright sepals, making the flowers narrow and three united filaments with anthers vertically along the androphore in *C.humbertii*, but five free stamens (Fig. 9a) in the other two species (at least close together in young flowers of *C.sambiranenensis*); the pistillate flowers have the stigmas united into an upright cone with a narrow opening between them (Fig. 9b).

*Ciccacoodei* (Ralim. & Petra Hoffm.) R.W.Bouman and *C.cryptophila* (Comm. ex A.Juss.) R.W.Bouman, also in subgenus Menarda, have rather open staminate flowers with free stamens and the stigmas in the pistillate flowers are on a short style, but well-developed and spreading and clearly split halfway.

Unlike in the previous two groups, the switch to moth pollination resulted in less distinct differences in the flowers; therefore, the moth- and non-moth-pollinated species had not been divided into separate genera. In comparison to the former two groups, *C.betsileani*, *C.coodei* and *C.cryptophila* conform in their morphology to non-moth-pollinated with open flowers with either well-developed stigmas or free, spreading stamens. *Ciccacryptophila* is in the basal clade of subgenus Menarda (Bouman et al. 2022: suppl. fig. 1). The other three species, *C.humbertii*, *C.perrieri* and *C.sambiranensis*, are in higher clades. Their pistillate flowers are similar to those of the moth-pollinated subgenus Glochidion in the cone-like united stigmas. Their staminate flowers have narrow, stiff sepals, but differ in the degree of connation of the stamens.

As few species could be observed, it is not possible to show where in the *Cicca* group moth pollination started, but likely between the basal group (with *C.cryptophila*) and the upper clades (which partly form a trichotomy).

Due to the high similarity with the pistillate flowers of *Glochidion*, several of the species in *Cicca* were formerly described or transferred to *Glochidion* (see Leandri (1937); Hoffmann and McPherson (2003)), but generally differ in the presence of disc glands, which are absent in *Glochidion* (Table 2).

### ﻿DendrophyllanthussectionDendrophyllanthus (Group 4 in Fig. 1A, Table 2)

With this group, *Ciccabetsileani* can serve as a non-moth-pollinated outgroup; see the previous group for a short description.

**Figure 10.**
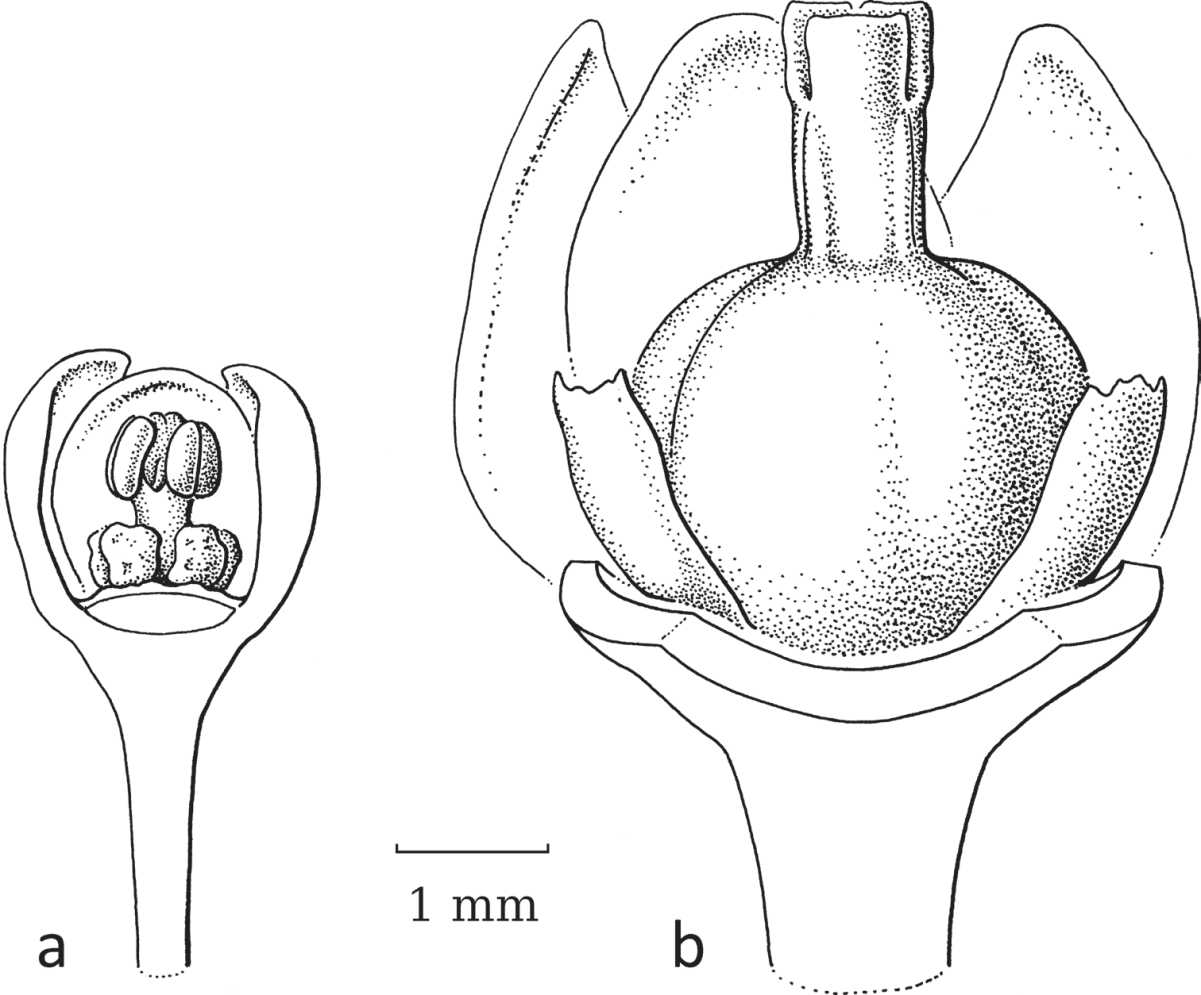
*Dendrophyllanthusclamboides* (F.Muell.) R.W.Bouman **a** staminate flower with part of sepals removed showing disc glands and united stamens **b** pistillate flower with part of sepals removed showing disc glands and united stigmas (**a***Carr 15875***b***NGF* (*Vandenberg*, *Womersley & Galore*) *42044*; both L). Illustration by Esmée Winkel, 2021.

**Figure 11.**
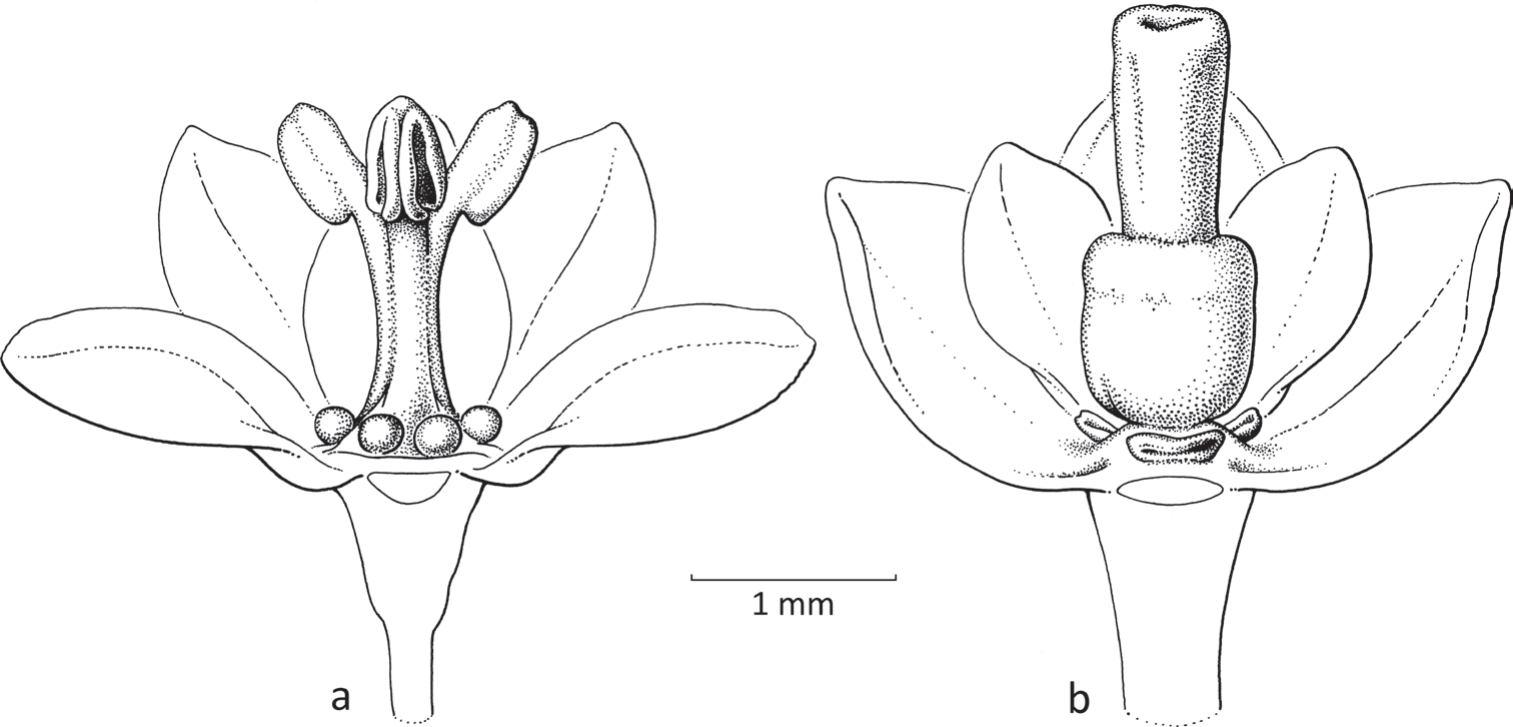
*Dendrophyllanthusglochidioides* (Elmer) R.W.Bouman **a** staminate flower with part of sepals removed showing disc glands and partly united stamens **b** pistillate flower with part of sepals removed showing disc glands and united stigmas (**a***PNH* (*Edaño*) *40177***b***PPI* (*Stone* et al.) *24*; both L). Illustration by Esmée Winkel, 2021.

**Figure 12.**
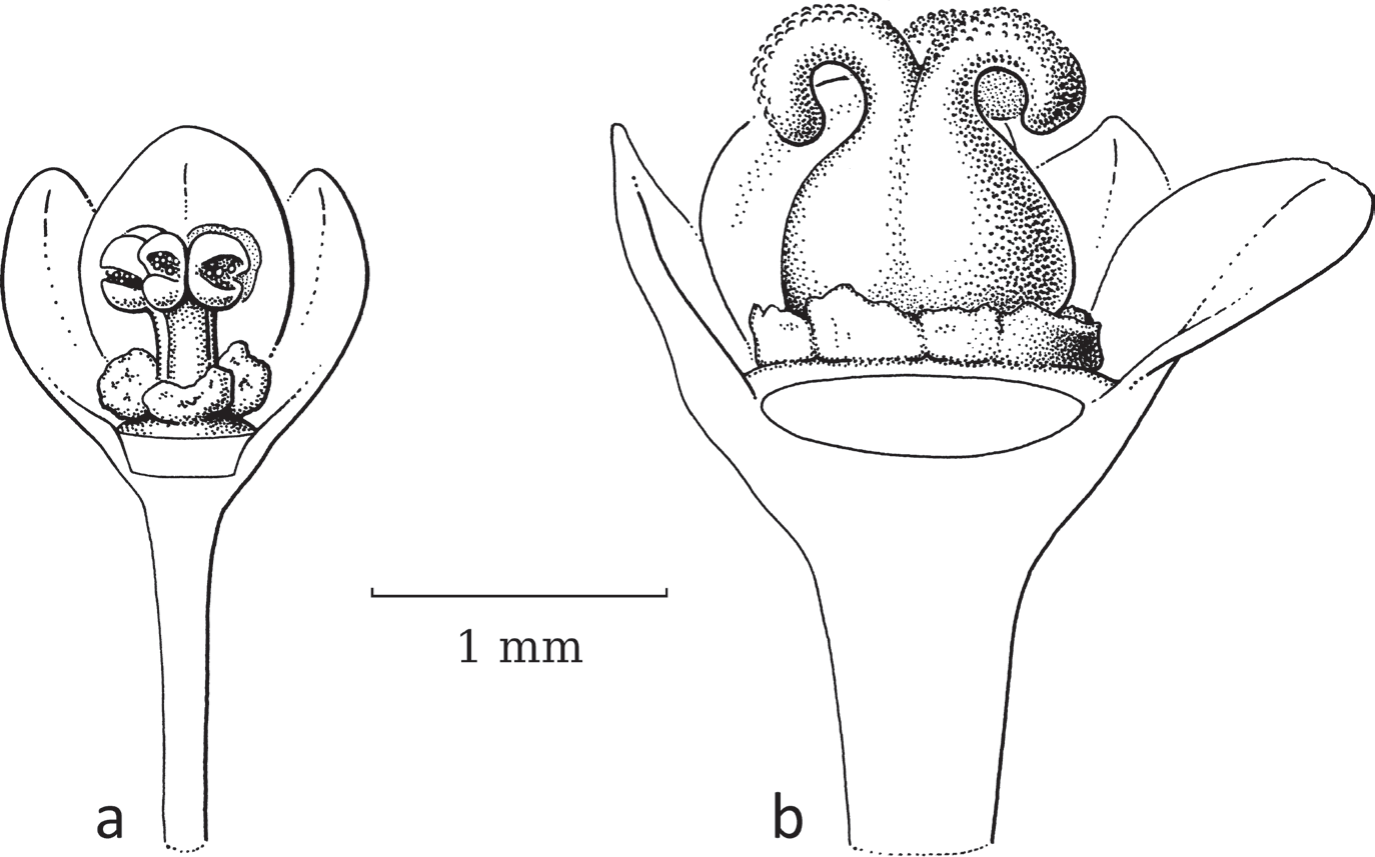
*Dendrophyllanthuswilkesianus* (Müll.Arg.) R.W.Bouman **a** staminate flower with partly removed sepals showing disc glands and the united stamens with small anthers with horizontal slits **b** pistillate flower with part of sepals removed, showing disc glands and well-developed, free, recurved, papillate stigmas (**a***A.C. Smith 6294***b***A.C. Smith 9630*; both L). Illustration by Esmée Winkel, 2021.

DendrophyllanthusS.Mooresect.Dendrophyllanthus is a New Caledonian group that comprises moth- and non-moth-pollinated species (Table 2) based on the comparison with the outgroup. However, the staminate flowers were often not seen or were young and in bud. They do not present a clear picture of morphological adaptations. The stamens are generally erect and free to completely united (Table 2). The pistillate flowers show either large, well-developed stigmas, generally free and sometimes recurved, or the stigmas are erect, free or united, sometimes small, in a tight upright circle with the stigmatic tissue on the inside (Table 2). *Dendrophyllanthusclamboides* (F.Muell.) R.W.Bouman (Fig. 10) shows united stamens and stigmas and is likely moth-pollinated like *D.glochidioides* (Elmer) R.W.Bouman with partly free stamens (Fig. 11). The interpretation that united stamens and stigmas are indicative of moth pollination is not necessarily always correct. *Dendrophyllanthusbuxoides* (Guillaumin) R.W.Bouman and *D.caudatus* (Müll.Arg.) R.W.Bouman are recorded by Kawakita and Kato (2004a) to be moth-pollinated, but their sturdy stigmas point at the opposite conclusion, though the stigmas are more or less upright (slightly spreading) and, in case of *D.caudatus*, bent inwards towards each other (only seen in young fruit). *Dendrophyllanthuswilkesianus* (Müll.Arg.) R.W.Bouman (Fig. 12) shows more or less united stamens (but small anthers with horizontal slits), but free, recurved, papillate stigmas and likely is not moth-pollinated.

Unfortunately, the published phylogenies for this group either only contain moth-pollinated species (Kawakita and Kato (2009), as Phyllanthussubgen.Gomphidiumsect.Adenoglochidion) or show a basal polytomy (Bouman et al. 2021). This means that it is uncertain where moth pollination started in the phylogeny and if there are species reversing to non-moth pollination. Additionally, due to lack of material, not all species mentioned by Kawakita and Kato (2004a, 2009) could be analysed.

### ﻿DendrophyllanthussectionLeptonema (Group 5 in Fig. 1A; Table 2)

DendrophyllanthusS.Mooresect.Leptonema (Baill.) R.W.Bouman is an Australian–New Caledonian group that shows the same developments as the previous group. Again, *C.betsileani*, which has free stigmas, can serve as the outgroup. The staminate flowers do not show any obvious morphological adaptations to moth-pollination and the pistillate flowers show either well-developed spreading stigmas or cone-like, united or free, upright stigmas (Table 2). Thus, some of the species in this group may already have adapted to moth pollination, some not. Kawakita and Kato (2004a, as Phyllanthussubgen.Gomphidiumsect.Gomphidium) listed a number of moth-pollinated species and three non-moth-pollinated species. The overlap with the species investigated here is very small with only two moth-pollinated species, of which *D.aeneus* (Baill.) R.W.Bouman has pistillate flowers that are adapted to moth pollination, but *D.vulcani* (Guillaumin.) R.W.Bouman seemingly has not (yet) adapted morphologically as it has well-developed, spreading stigmas.

**Figure 13.**
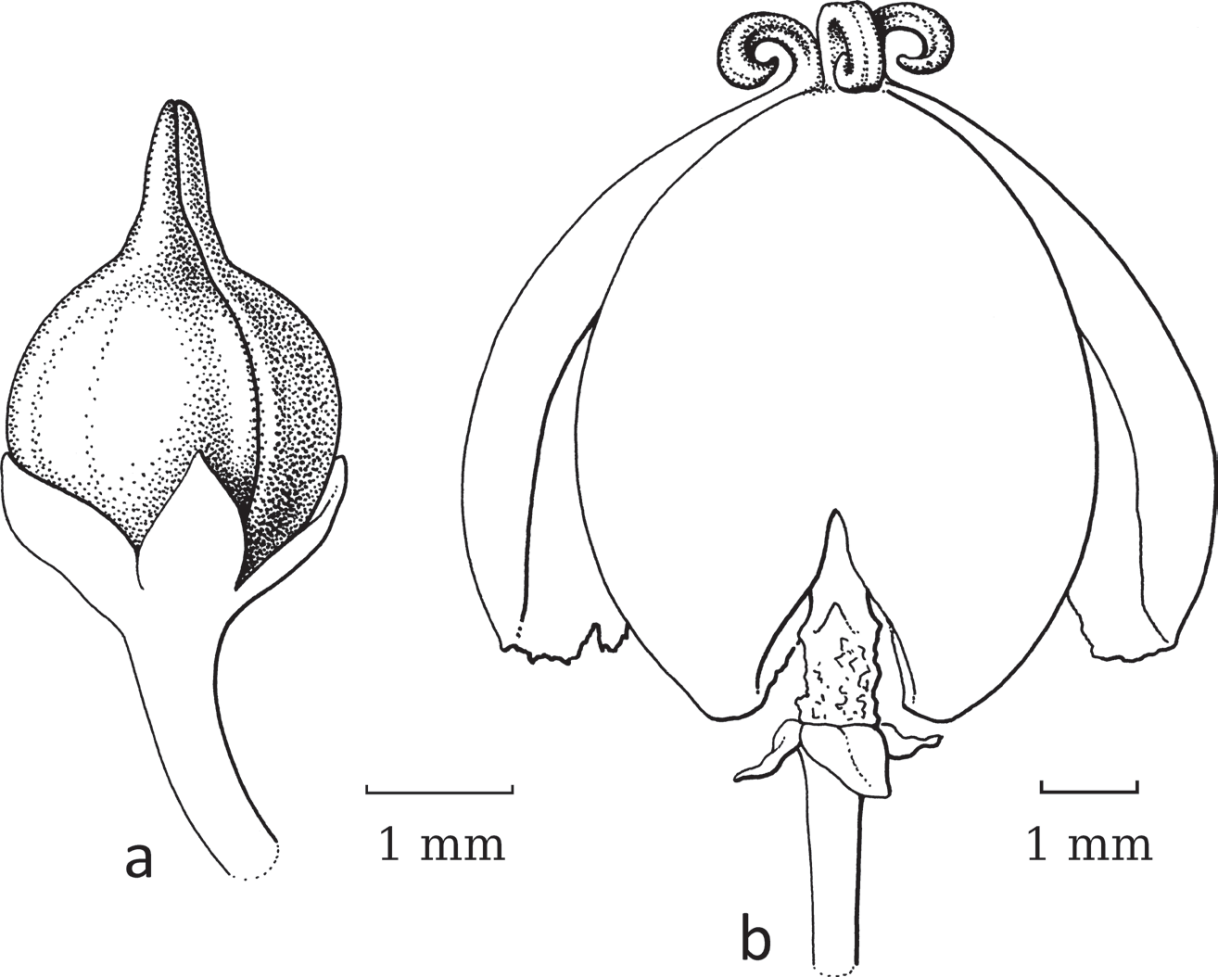
*Dendrophyllanthusligustrifolius* (S.Moore) R.W.Bouman **a** developing fruit with stigmas erect and closed **b** dehiscing fruit with non-papillate stigmas recurved (**a***MacKee 19634***b***McPherson 5025*; both L). Illustration by Esmée Winkel, 2021.

**Figure 14.**
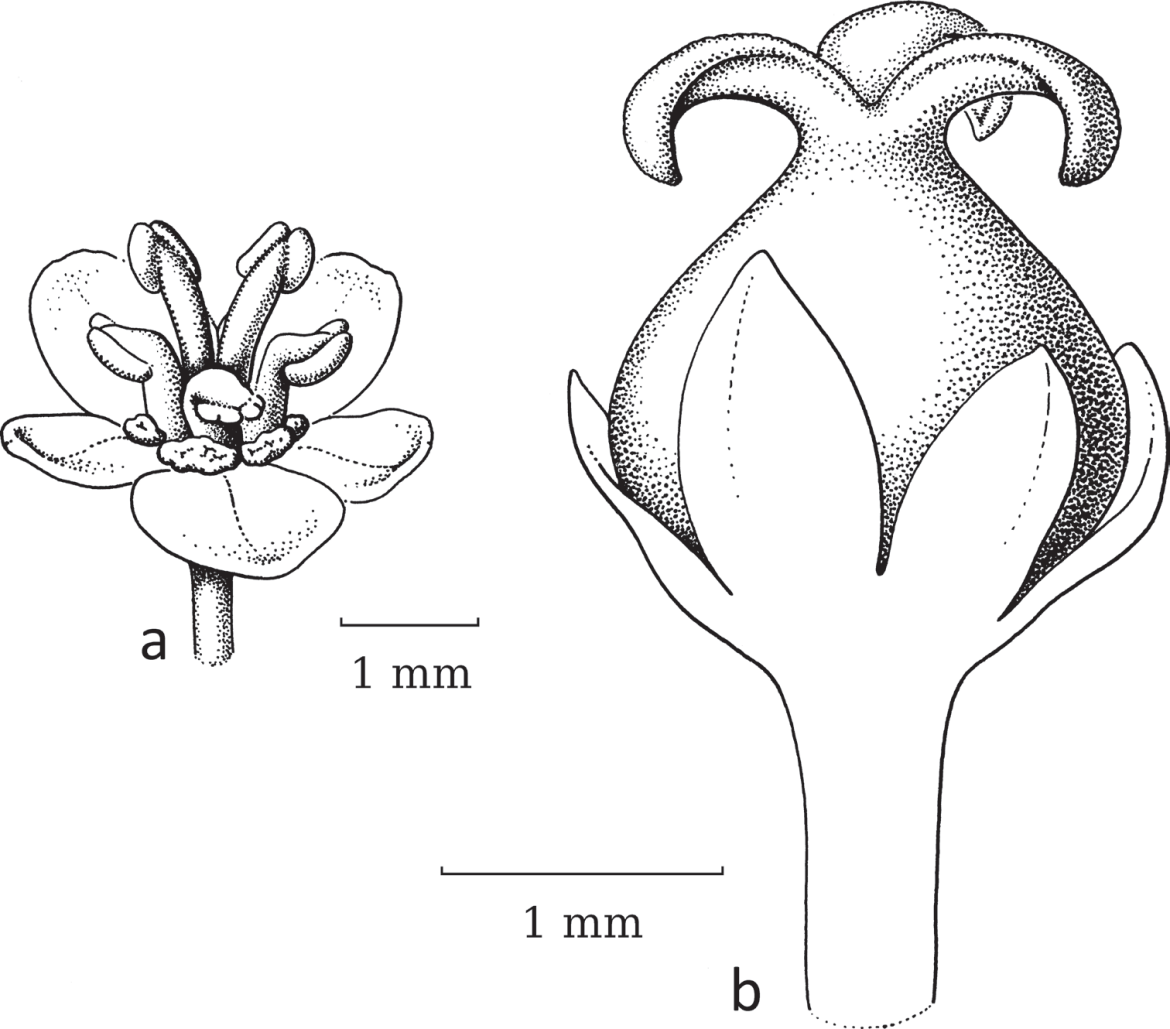
*Dendrophyllanthushypospodius* (F.Muell.) R.W.Bouman **a** staminate flower with free stamens **b** pistillate flower with free, recurved stigmas (*Bruhl et al. 1123*, L). Illustration by Esmée Winkel, 2021.

The phylogeny of Kawakita and Kato (2009) included only moth-pollinated species, whereas the phylogeny of Bouman et al. (2021) showed that, perhaps in part of the clade, with *D.ligustrifolius* (S.Moore) R.W.Bouman at the base, a switch to moth pollination occurred, because *D.ligustrifolius* likely is moth-pollinated (Fig. 13) and *D.hypospodius* (F.Muell.) R.W.Bouman (Fig. 14) and *D.serpentinus* (S.Moore) R.W.Bouman likely are not moth-pollinated. Unfortunately, the upper part of the clade comprises a few polytomies and weakly-supported branches, thus this may change in the future when more species and/or markers are added. In this group, *D.bupleuroides* (Baill.) R.W.Bouman, *D.kanalensis* (Baill.) R.W.Bouman and *D.loranthoides* (Baill.) R.W.Bouman are probably not moth-pollinated; *D.vulcani* is disputable (it seems to have moth pollination (Kawakita and Kato 2004a, 2009), but is morphologically not adapted); *D.aeneus*, *D.guillauminii* (Däniker) R.W.Bouman and *D.ligustrifolius* have moth-pollination; and the pollination of *D.favieri* (M.Schmid) R.W.Bouman and *D.unifoliatus* (M.Schmid) R.W.Bouman is unknown.

### ﻿KirganeliaA.Juss.subgenusKirganelia section KirganeliaversussectionPseudomenarda (Group 6 in Fig. 1A; Table 2)

In *Kirganelia*, section Pseudomenarda (Müll.Arg.) R.W.Bouman is the non-moth-pollinated outgroup to section Kirganelia, where moth pollination is recorded. Section Pseudomenarda is a small taxon with two species, of which *K.somalensis* (Hutch.) R.W.Bouman (Fig. 16) was seen. It shows staminate flowers of 2.5–3 mm in diameter with five sepals, five disc lobes, two outer free and three inner united stamens. The pistillate flowers are very open, ca. 3.5 mm in diameter, with five relatively large sepals, a broad, ring-like disc and a 5-locular (can be 3-locular; see Hutchinson (1912)) ovary with a terminal, ca. 0.3 mm long style and then five horizontally spreading stigmas ca. 1 mm long, split in the upper third.

**Figure 15.**
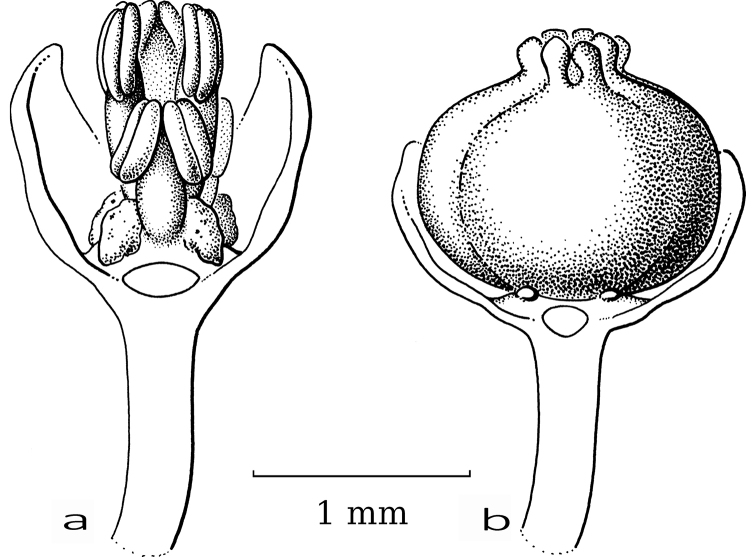
*Kirganeliareticulata* (Poir.) Baill. **a** staminate flower with part of sepals removed, showing disc glands and two layers of united stamens **b** pistillate flower with part of sepals removed showing small disc glands and small, upright, split free stigmas bent towards each other (**a***Maxwell 91-1025***b***Put 2297*; all L). Illustration by Esmée Winkel, 2021.

Section Kirganelia, *Kirganeliareticulata* (Poir.) Baill. was reported to be moth-pollinated (Kawakita et al. 2015; Fig. 15). The staminate flowers are very small and inconspicuous (ca. 2 mm in diameter) with five or six sepals in two whorls, five or six small disc glands and five or six stamens in two very tight whorls, outer two or three free, inner three with filaments basally connate; these staminate flowers are usually found unopened in dried specimens. The pistillate flowers have the same number of sepals and disc lobes as the staminate flowers, the ovary is small with four or five short stigmas, slightly split apically, bent towards each other and forming a circular cone that is open on the inside. Kawakita et al. (2015) reported that six moth species visit the flowers of *K.reticulata*, of which three have become parasitic again, causing the ovules/fruits to gall and one species also induces the fruit to become inflated. A possible reason for the reversal to parasitism is protection against predation by specialised braconid wasps (Kawakita et al. 2015). Predation by the wasps is likely as *K.reticulata* has relatively small fruits (ca. 4.5 mm in diameter), thus long ovipositors are not needed.

**Figure 16.**
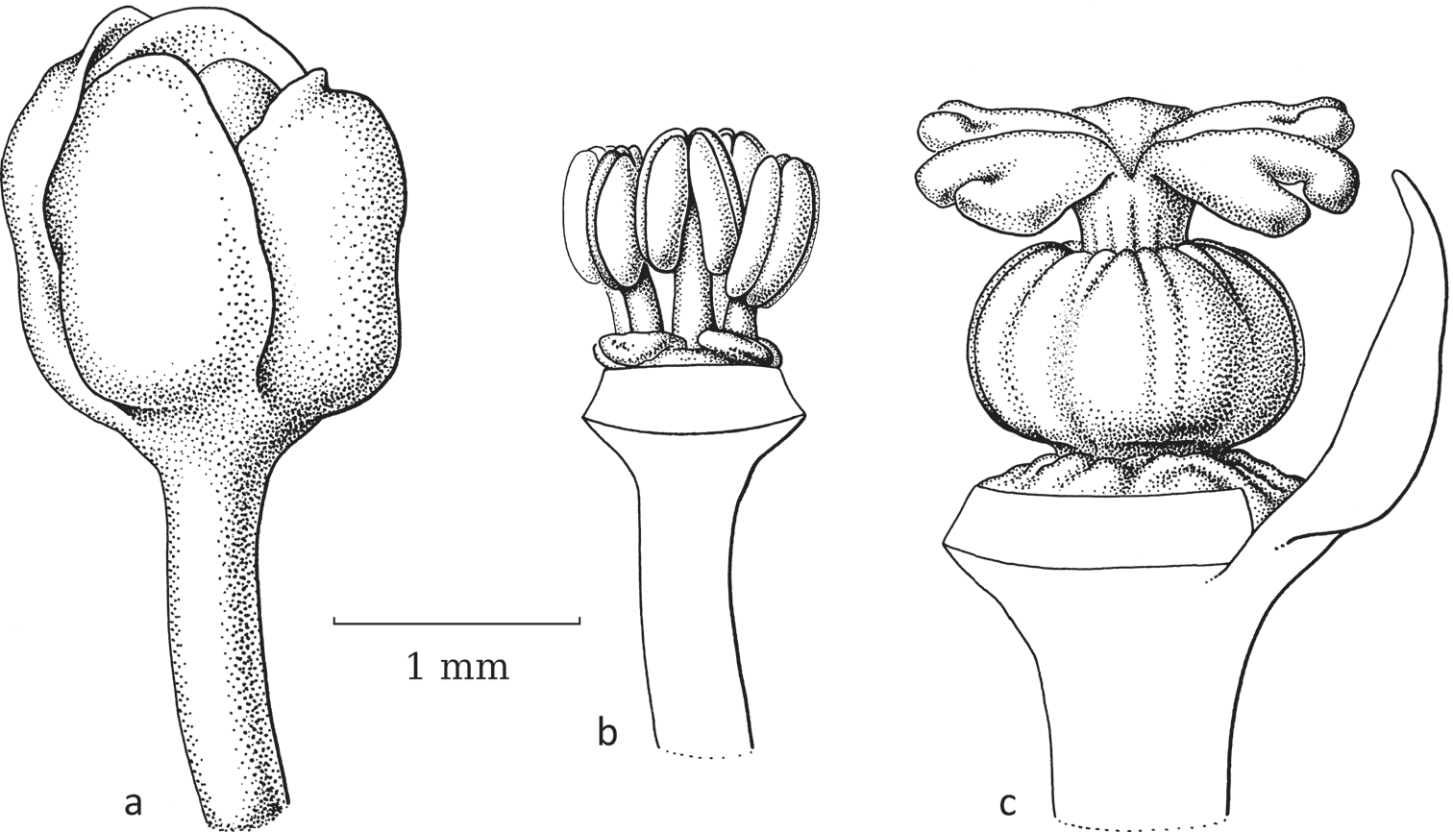
*Kirganeliasomalensis* (Hutch.) R.W.Bouman **a** staminate flower, closed **b** staminate flower with part of sepals removed showing disc glands and free stamens **c** pistillate flower with part of sepals removed showing flat, circular and indistinct disc and well-developed, horizontal free stigmas (*Kilian 1718 & Lobin 6572*, WAG). Illustration by Esmée Winkel, 2021.

Adaptation in flower morphology of *K.reticulata* for moth pollination is seemingly only visible in the pistillate flowers; the staminate flowers with tightly, vertically grouped stamens are, at most, smaller than those in other species and then less accessible for various groups of insects. As in BreyniasectionBreynia, the stigmas in the pistillate flowers are shorter and, as in *Glochidion*, the stigmas are united, upright and short, forming an erect ring on top of the ovary and forming a depression inside. In contrast, in section Pseudomenarda, the stigmas are still well-developed and spreading. Additionally, some species within the genus, belonging to the now-subsumed group PhyllanthussectionHemicicca (Baill.) Müll.Arg., possess different flower features; especially *K.flexuosa* (Siebold & Zucc.) R.W.Bouman, which has staminate flowers with reddish, spreading sepals and two free stamens (see Kato and Kawakita (2017)). Their pollinators are unknown.

## ﻿Discussion

### ﻿Palaeotropics

As a result of obligate leafflower moth pollination, adaptations in the morphology of staminate and pistillate flowers were expected because they can improve pollination efficiency and the fit with the moths, while also optimising pollen uptake, pollen deposition and oviposition for the pollinators. However, because the mutualistic relationship has evolved several times in tribe Phyllantheae, there could be differences in morphological adaptations or similar morphological patterns in the different groups could have arisen through convergent evolution. Recent studies have shown that the mutualism between leafflowers and leafflower moths is more complex than first described (Kato et al. 2003) as there are differences in the level of species specificity (Kawakita and Kato 2004a, 2006; Zhang et al. 2012; Li et al. 2015; Yang and Li 2018), cheaters of the mutualism (Kawakita et al. 2015; Wang et al. 2020) and defences against this antagonistic relationship (Goto et al. 2010; Furukawa and Kawakita 2017). Furthermore, possible reversals in flowers are indicated here.

Flowers associated with the obligate moth pollination mutualism show, as a visual stimulus to attract the nocturnally active *Epicephala* moths, a contrast between the light (green to yellowish) sepals and the dark night sky, but this is also observed in non-moth-pollinated flowers. Olfactory cues were detected in a number of studies, differences that likely promoted speciation as they differed either in strength or composition between the sexes (Svensson et al. 2010; Okamoto et al. 2013; Okamoto 2017; Huang et al. 2015; Zhang et al. 2016). Okamoto et al. (2007; see also Okamoto (2017)) showed that GlochidionsubgenusGlochidion produces floral odours, often typical of each species, but different between flower sexes, that attract the female moths. Additionally, *Breyniavitis-idaea* (Burm.f.) C.E.C.Fisch. produces two floral odours. Attractants are likely crucial, especially during the nocturnal pollination and differences in smell between the sexes can help guide the moth first to the staminate and later the pistillate flowers. Therefore, nectar glands like the disc or disc glands may still be as important as the tissue to emit the odours; the moths themselves do not seek nectar and the plants likely do not attract other insects. The glands/discs are present in most Phyllantheae, except in GlochidionsubgenusGlochidion and *Breynia*; in these taxa, perhaps the receptacle produces the odour. The two other subgenera of *Glochidion* (*Pseudoactephila* and *Phyllanthodendron*) have disc glands in the flowers, but these have become almost petal-like and likely are not producing nectar any longer.

The majority of the moth-pollinated flowers are small with no bright colours, while the narrow shape of many is geared more towards a mechanical fit to the pollinator. The adaptations in staminate flowers that seem most common are a tight, cylindrical ring of sepals, with united, or at least upright, stamens with the anthers vertically along the androphore (opening towards the sepals) or with the anthers at the top of the androphore. This is especially demonstrated in BreyniasectionBreynia and GlochidionsubgenusGlochidion (groups 1 and 2, respectively). With herbarium material, it is difficult to access the tightness of the sepals; therefore, this is often not noted, except when the sepals are thick and upright like several species in CiccasubgenusMenarda (group 3), *Pyllanthusgraveolens* (PhyllanthussubgenusConami; group 7) and several species in PhyllanthussubgenusMicroglochidion (Group 8). If the staminate flowers produce a tight calyx cylinder, then as soon as the moth probes with its proboscis, covered with sensilla, the sensilla become dusted with pollen. This seemingly is a very effective mechanism.

Female moths have to transfer the pollen to the pistillate flowers, which is generally facilitated by the plant as most Phyllantheae species are monoecious and may have fascicles with both sexes (often many-flowered in *Glochidion*), whereby the sexes can be receptive at different moments, or the sexes are separated in space on one branch with the staminate flowers proximally and the pistillate flowers distally (often in *Breynia* and *Phyllanthus* s.l.). A few species are reported as dioecious, but this is not easy to judge from herbarium specimens; it can also be the result of dichogamy (an extended separation in time as the staminate flowers usually develop later than the pistillate flowers). For the placements of the different sexes along branches in the various species in Thailand, see for *Breynia*: van Welzen and Esser in van Welzen (2007), for *Glochidion*: van Welzen (2007) and for *Phyllanthus* s.l.: Chantaranothai (2007).

In the pistillate flowers, the upright, closely set sepals and the reduced stigmatic surface may serve two functions, to facilitate the pollen transfer and to preclude visits by insects other than leafflower moths. Two reversals (*Breyniaretusa* and *Glochidionsericeum*) show that well accessible, completely developed and spreading stigmas no longer attract moths. A reduction in the style length and stigmas spreading was shown as an indicator of *Epicephala* pollination by Kawakita and Kato (2009: fig. 2). The reduction of the stigmatic tissue is achieved in two ways, either by shortening the stigmas or by folding the stigmas into a vertical (pyramidal) cone with a small, apical opening where the female moth can deposit the pollen. A reduction of the stigmas is seen in BreyniasectionBreynia. *Kirganeliareticulata* seems to follow both strategies: the stigmas are short and folded together. All other groups show cone-like united stigmas as in *Glochidion*. Several moth-pollinated South American species of *Phyllanthus* may still have well-developed, spreading stigmas (Kawakita et al. 2019; see below). Additionally, the stigmatic papillae seem to disappear in the moth-pollinated species; compare Fig. 3 (*Breynialithophila* Welzen & Pruesapan, not moth-pollinated) with Fig. 4 (*Breyniaglauca* Craib, moth-pollinated).

The taxa included here have been shown by other authors to be pollinated and/or parasitised by *Epicehala*. However, several taxa display similar morphological adaptations to those treated here and future studies might uncover new instances of plants in tribe Phyllantheae in a mutualistic relationship with *Epicephala* moths. The main pollination system in *Nymphanthus* Lour. has not yet been uncovered. Kawakita and Kato (2009) reported miscellaneous observations of dipterans visiting the typical cross-shaped flowers, but this has not been confirmed. However, species of N.sectionScepasma (Blume) R.W.Bouman have very different flowers with erect sepals and *N.lamprophyllus* (Müll.Arg.) R.W.Bouman, for example, has a long style with small stigma lobes at the top. Similarly, pistillate flowers of *Emblicarufuschaneyi* (Welzen, R.W.Bouman & Ent) R.W.Bouman also have a long stylar column and a reduced disc (Bouman et al. 2018). Other species of *Emblica* usually have well-developed spreading stigmas, but whether they are associated with *Epicephala* is not well known.

Adaptation to moth pollination seems to be comparatively more pronounced in the Palaeotropics (and Pacific islands) in what was formerly known as *Breynia* (B.subgen.BreyniasectionBreynia) and *Glochidion* (G.subgenusGlochidion) (Kawakita et al. 2019). It is often speculated that the obligate moth pollination contributed to a proliferation in species (Kato et al. 2003; Kawakita and Kato 2004a), especially in *Glochidion* (> 300 spp.) and New Caledonian *Dendrophyllanthus* (~ 160 spp.), which are speciose groups that have radiated relatively recently (see Kawakita and Kato (2009)). *Breynia* is somewhat less species rich, thus other factors than moth pollination likely contributed to the high speciation rate seen in these groups. The olfactory attractants may play a role (see above), but also the greater variation in flower shapes in *Glochidion* and *Dendrophyllanthus*.

In conclusion, these Palaeotropical groups show some general trends in morphological adaptation in flowers resulting from obligate moth pollination:

- Sepals are usually stiff and upright and in a tight group around either the stamens or the ovary.
- Disc and disc glands are reduced.
- Stamens are united with the anthers upright along the androphore.
- Stigmatic surface is reduced, either by reducing the length of the stigmas to short stigmas or by uniting them into a cone with an apical round opening.
- Likely stigma papillae disappear.

However, these trends are not always completely present, for example, mutualistic relationships were found in species with well-developed stigmas (see some discussions above and Table 2).

### ﻿Neotropics

**Figure 17.**
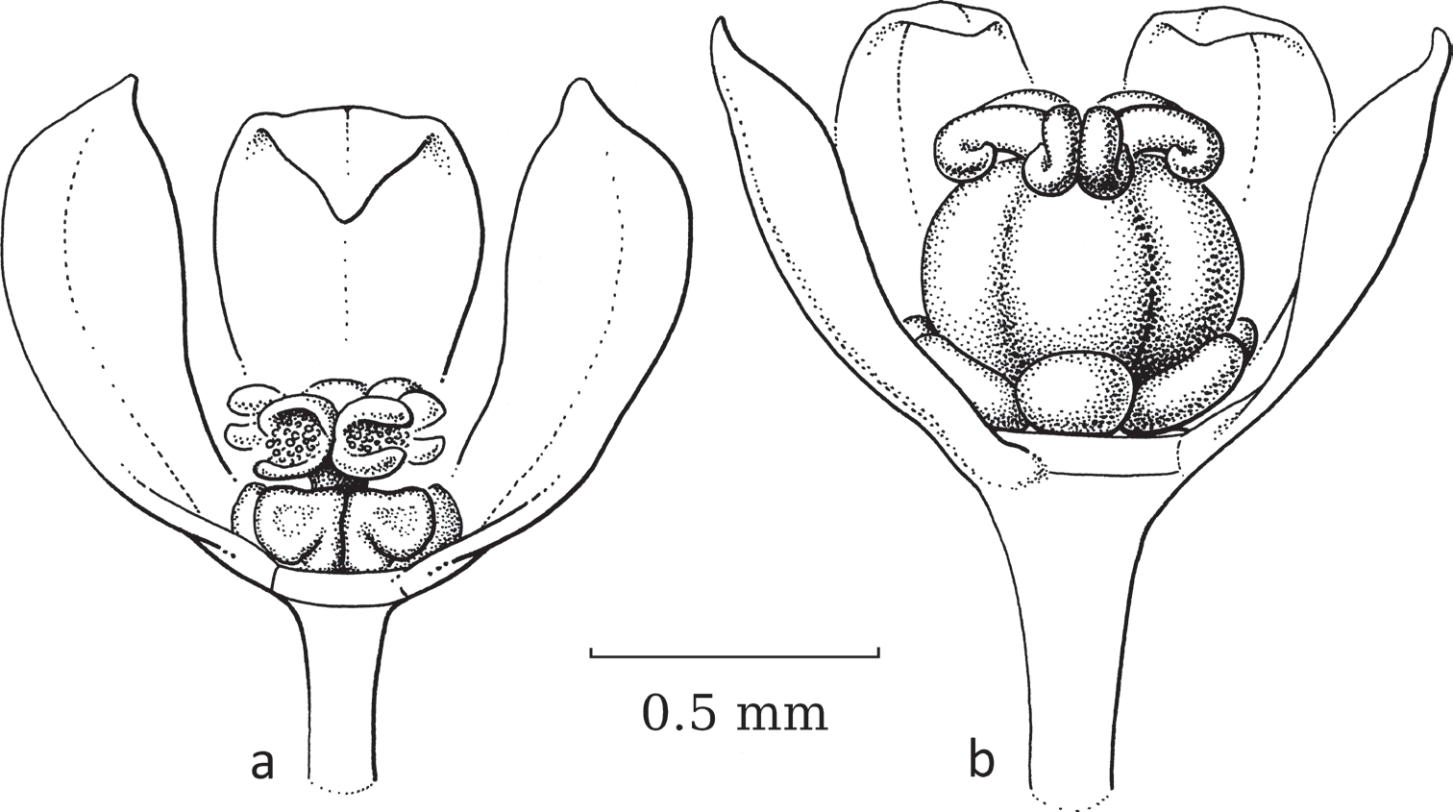
*Phyllanthusorbiculatus* Rich **a** staminate flower with part of sepals removed, showing large disc gland and united stamens with small anthers with horizontal slits **b** pistillate flower with part of sepals removed showing large disc glands and well-developed free, horizontal to recurved stigmas (*Herzog 1314*, L). Illustration by Esmée Winkel, 2021.

Based on the morphological trends found in the Palaeotropics, the situation in the Americas is evaluated here. Moth pollination in the Neotropics was recently reported by Kawakita et al. (2019). The moth *Epicephalachancapiedra* Kawakita & Kato, 2019, was found on three herbaceous species: *Moerorisamara* (Schumach. & Thonn.) R.W.Bouman, *M.stipulata* Raf. and *Phyllanthusorbiculatus* Rich. (Fig. 17). These three herbaceous species are pollinated by ants and as female *E.chancapiedra* does not have sensilla on the proboscis, the moths are (secondary) parasites, only laying eggs in the pistillate flowers without active pollination (Kawakita et al. 2019). The flowers of both sexes in these three species are small, hanging, quite open and the stigmas are generally short, but well-developed and spreading. Only the short stigmas might be an adaptation to moths, but it is more likely an adaptation to the ants or no pollination is needed as all pistillate flowers develop fruits (also in a hothouse without pollinators, but with limited seed production, pers. observ.).

In the genus PhyllanthusL.subgenusCiccastrum (Müll.Arg.) R.W.Bouman (Fig. 1B) can act as a local outgroup for comparisons. This group consists of two species, *P.purpusii* Brandegee (Fig. 19) and *P.riedelianus* Müll.Arg. Both species have free stigmas that recurve over the ovary (*P.purpusii*) or that are bent towards each other in fruit (*P.riedelianus*). Only the staminate flowers of *P.purpusii*, each consisting of a narrow and tight cylinder of stiff upright sepals and three united stamens with the anthers apically along the androphore (like Breyniasect.Breynia), may point at moth pollination. The staminate flowers of *P.riedelianus* show no distinct adaptations to moth pollination (more like Breyniasubg.Sauropus). However, *P.riedelianus* (not in the phylogeny of Bouman et al. (2021)) is doubtfully placed in this group and is strongly divergent from *P.purpusii* (Webster, 2002); see Torres et al. (2020).

**Figure 18.**
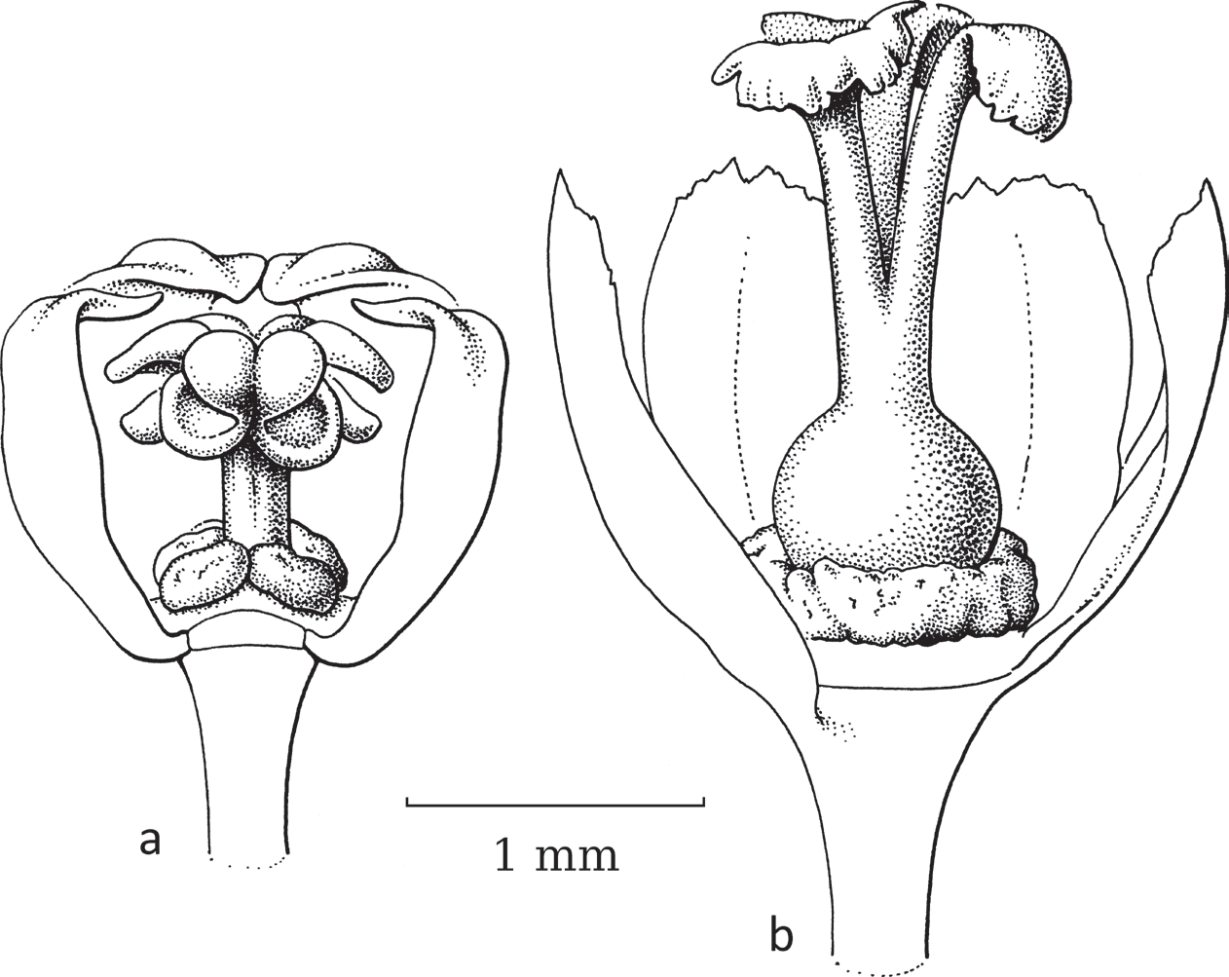
*Phyllanthussalviifolius* Kunth **a** staminate flower with part of sepals removed showing disc glands and three united stamens with free small anthers with horizontal slits **b** pistillate flower with part of sepals removed showing disc glands and upright stigmas recurved at the apex (*Breteler 3312*, L). Illustration by Esmée Winkel, 2021.

Kawakita et al. (2019) also looked for moth pollination in four woody species in the subgenera *Conami* (Aubl.) G.L.Webster and *Xylophylla* (L.) Pers. of *Phyllanthus*: *P.acuminatus* Vahl, *P.graveolens* Kunth, *P.huallagensis* Standl. ex Croizat and *P.salviifolius* Kunth (Fig. 18). In all cases, the stigmas do not show reduced surfaces and the stamens are often not upright (except more or less in *P.graveolens*) and are, thus, usually not readily accessible to moths. The pollination relationship does not appear to be obligate as other pollinators are present (gall midges and thrips; Kawakita et al. (2019)), which may mean that the relationship with the moths is rather recent and perhaps still developing and morphological adaptations are still lacking.

**Figure 19.**
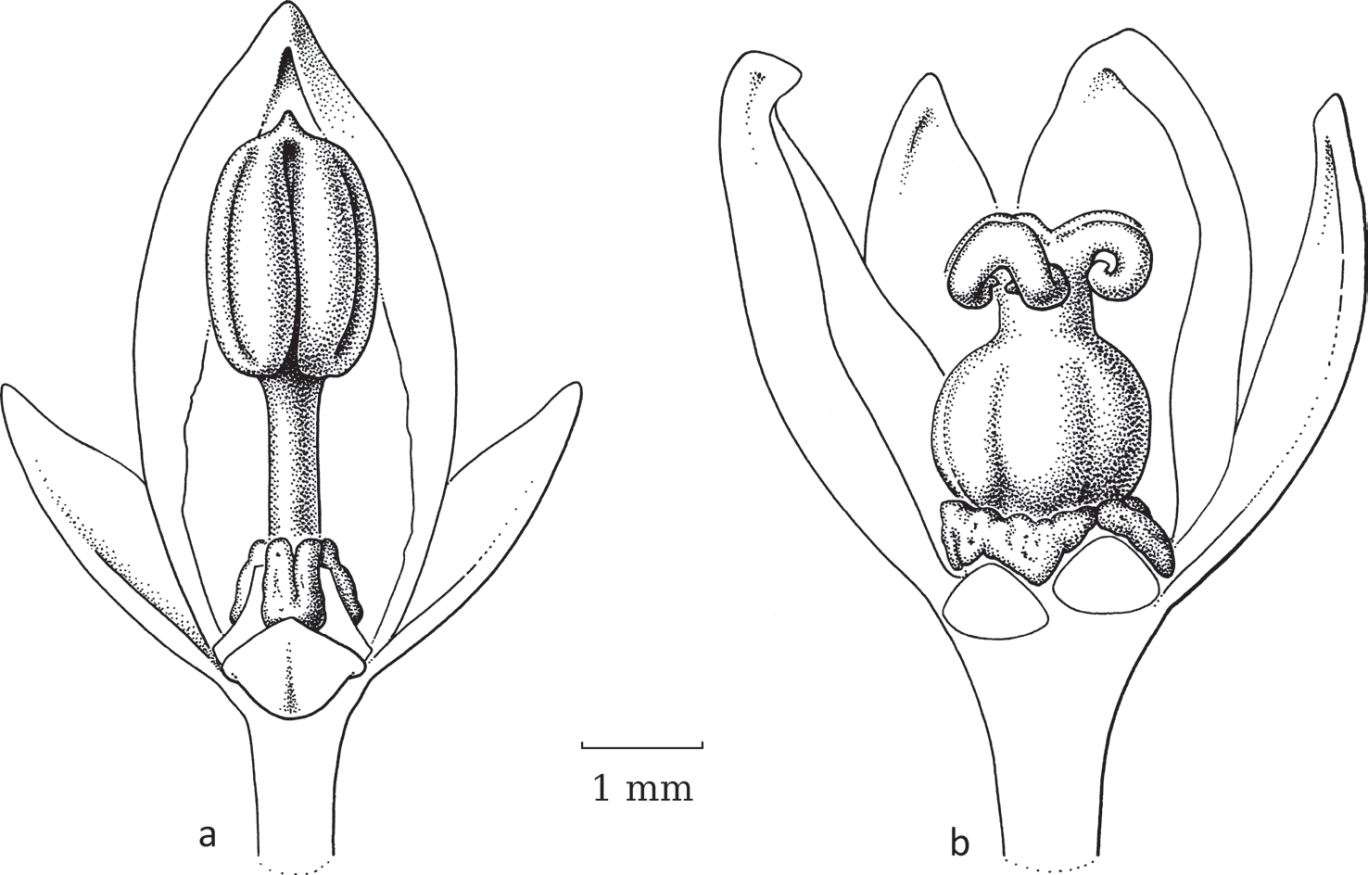
*Phyllanthuspurpusii* Brandegee **a** staminate flower with sepal removed showing disc glands and three united erect stamens with the anthers along the androphore **b** pistillate flower with sepal removed showing circular disc and spreading stigmas (*Quarles van Ufford 266*, U). Illustration by Esmée Winkel, 2021.

**Figure 20.**
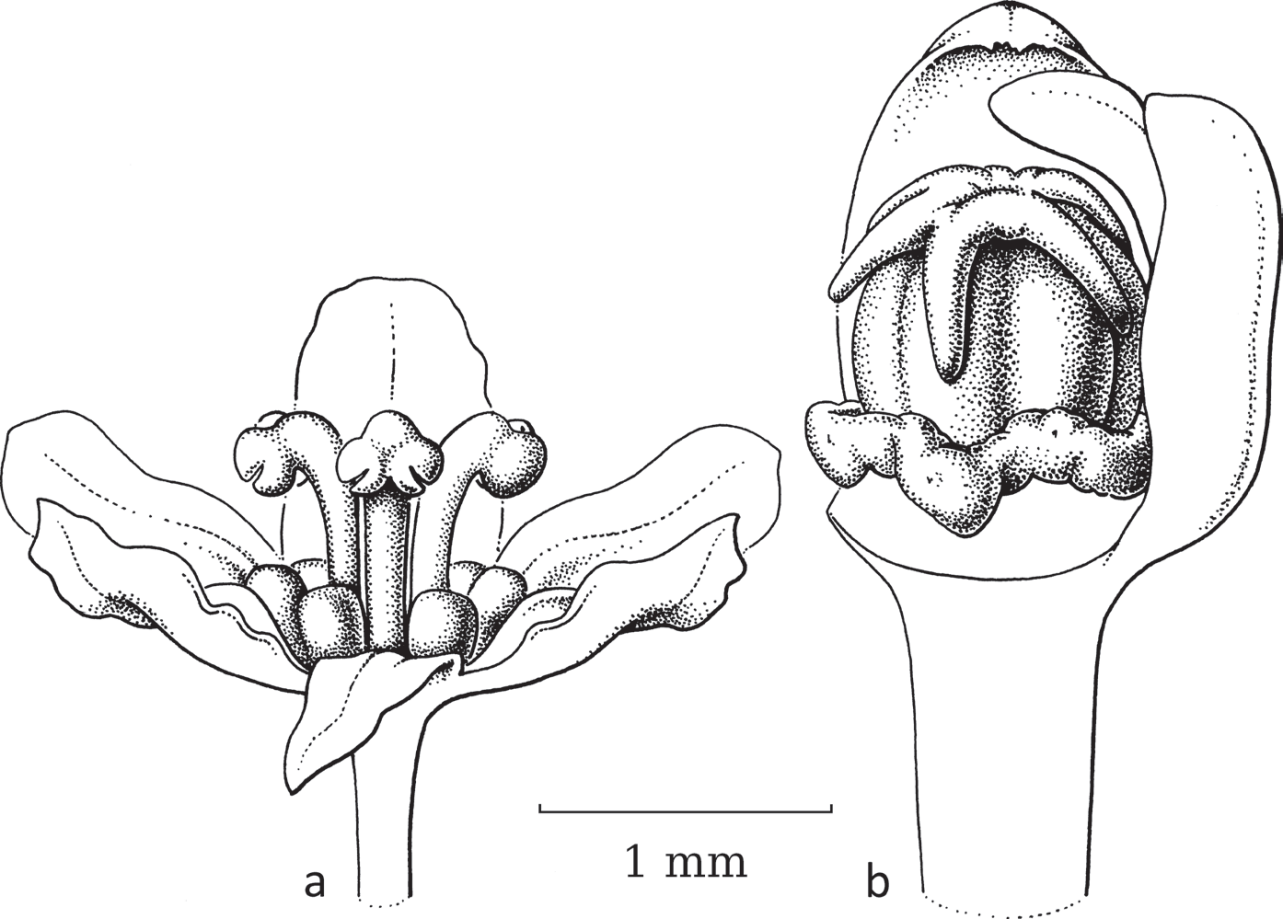
*Phyllanthusmyrsinites* Kunth **a** Staminate flower with part of sepals removed showing disc glands and free stamens **b** pistillate flower with part of sepals removed showing ring-like disc and well-developed free and recurved stigmas (*Wade Davis 14*, U). Illustration by Esmée Winkel, 2021.

**Figure 21.**
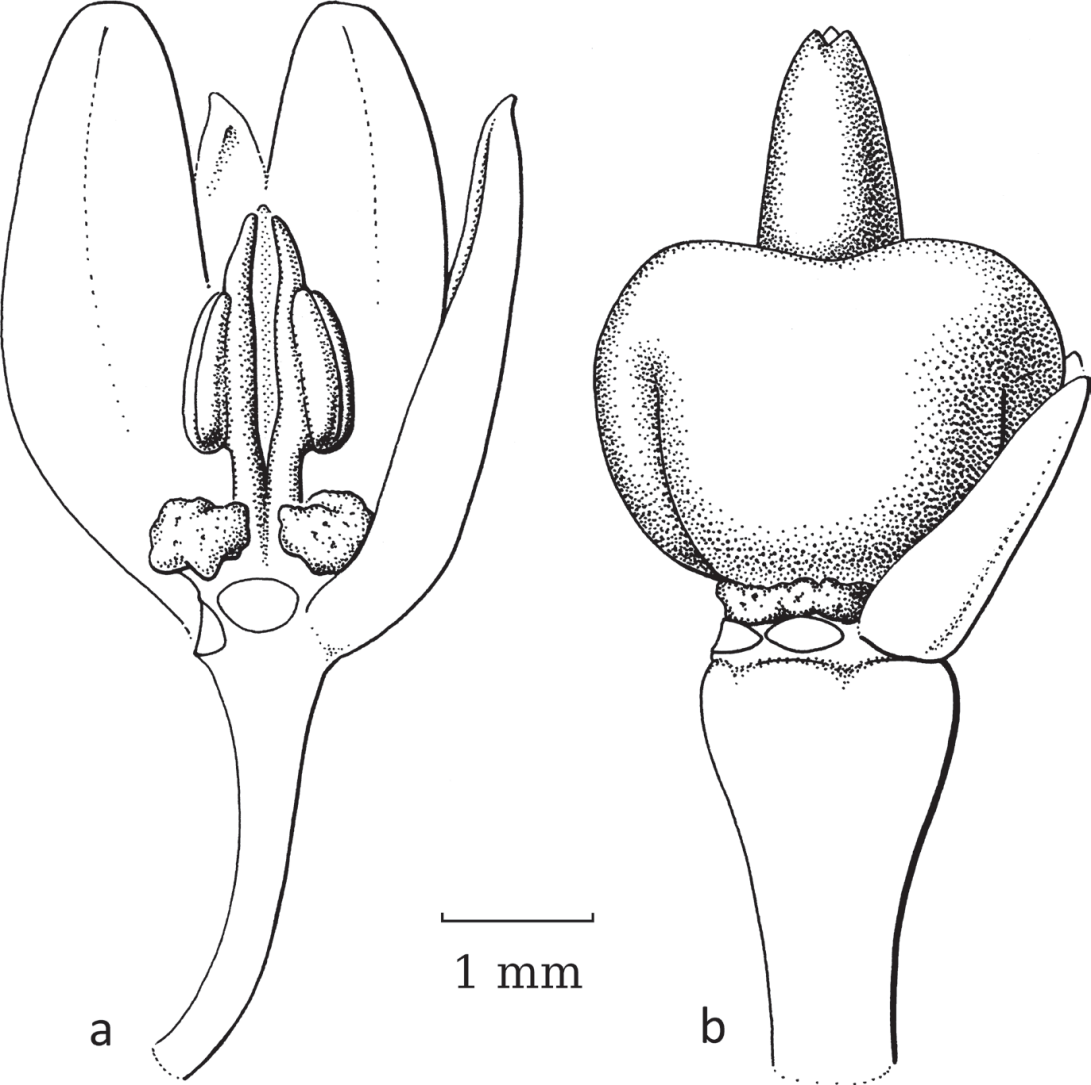
*Phyllanthusvacciniifolius* Müll.Arg. **a** staminate flower with part of sepals removed showing disc glands and three erect, free stamens with appendaged connectives **b** pistillate flower with part of sepals removed showing indistinct circular disc and upright, connate stigmas (*Prance 28343*, U). Illustration by Esmée Winkel, 2021.

Kawakita et al. (2019) mentioned PhyllanthusL.subgenusMicroglochidion (Müll.Arg.) Jean F.Brunel as a group with likely mutualistic pollination as there is some floral resemblance with *Glochidion*. Typical in the group is that the leaves often have a subapical extrafloral nectary abaxially, generally subapical above the mid-rib. In this group, as defined by Bouman et al. (2022), there are generally six sepals in two whorls; staminate flowers with six disc glands and three free, upright stamens; and pistillate flowers generally with a circular disc and a 3-locular ovary with the stigmas variously arranged. The situation is not completely clear as a large number of taxa could not be sampled. Not all taxa show visible morphological adaptations to moth pollination. *Phyllanthuslediformis* Jablonski, *P.majus* Steyerm., *P.myrsinites* Kunth (Fig. 20) and *P.neblinae* Jablonski have pistillate flowers with well-developed stigmas and thin sepals that generally are spreading and also have relatively large anthers and upright stamens. *Phyllanthusduidae* Gleason, *P.pycnophyllus* Müll.Arg. and *P.vacciniifolius* (Müll.Arg.) Müll.Arg. (Fig. 21) show an apical cone of united stigmas with three small lobes apically around a narrow opening, the typical situation as in GlochidionsubgenusGlochidion; their staminate flowers have upright stamens and high stiff, upright sepals. Very likely, the species with united stigmas are moth-pollinated as the staminate and pistillate flowers show adaptations like those in other groups: limited stigma surface and narrow staminate flowers. The phylogeny of Bouman et al. (2021) only shows one representative for this group (*P.vacciniifolius*); thus, it is not clear if a single adaptation to moth pollination occurred in an ancestral species or multiple adaptations.

PhyllanthusL.subgenusXylophylla (L.) Pers. section Epistylium (Sw.) Griseb. was another taxon mentioned by Kawakita et al. (2019) as a Central American (West Indies) group that might have mutualistic relationships with *Epicephala* moths. Herbarium material was largely lacking; we saw only a single sheet of *P.axillaris* with young pistillate buds (*Proctor 18349*, Jamaica, U), which seem to have a closed stigma cone as in Glochidionsubgen.Glochidion, which may be indicative of mutualistic pollination.

Thus, it appears that, in the Neotropics, the relationship with *Epicephala* moths probably is evolutionarily younger than in the Palaeotropics, because many plant species do not show strong adaptations to moth pollination and, as shown by Kawakita et al. (2019), several groups are likely not obligately moth-pollinated as other insect groups also pollinate the flowers. However, the pollination systems in the Neotropics are very understudied in comparison to those in the Palaeotropics.
